# Fibroblastic Reticular Cells From Lymph Nodes Attenuate T Cell Expansion by Producing Nitric Oxide

**DOI:** 10.1371/journal.pone.0027618

**Published:** 2011-11-14

**Authors:** Stefanie Siegert, Hsin-Ying Huang, Chen-Ying Yang, Leonardo Scarpellino, Lucie Carrie, Sarah Essex, Peter J. Nelson, Matthias Heikenwalder, Hans Acha-Orbea, Christopher D. Buckley, Benjamin J. Marsland, Dietmar Zehn, Sanjiv A. Luther

**Affiliations:** 1 Department of Biochemistry, University of Lausanne, Epalinges, Switzerland; 2 Swiss Vaccine Research Institute, and Centre Hospitalier Universitaire Vaudois (CHUV), Service of Immunology and Allergy, Lausanne, Switzerland; 3 School of Immunity and Infection, Institute for Biomedical Research, Medical Research Council Center for Immune Regulation, University of Birmingham, Birmingham, United Kingdom; 4 Medical Policlinic, Ludwig-Maximilians University/Helmholtz-Zentrum München, Munich, Germany; 5 Institute for Virology, Technical University Munich (TUM), Munich, Germany; 6 Service of Pneumology, Centre Hospitalier Universitaire Vaudois (CHUV), University of Lausanne, Lausanne, Switzerland; McGill University, Canada

## Abstract

Adaptive immune responses are initiated when T cells encounter antigen on dendritic cells (DC) in T zones of secondary lymphoid organs. T zones contain a 3-dimensional scaffold of fibroblastic reticular cells (FRC) but currently it is unclear how FRC influence T cell activation. Here we report that FRC lines and *ex vivo* FRC inhibit T cell proliferation but not differentiation. FRC share this feature with fibroblasts from non-lymphoid tissues as well as mesenchymal stromal cells. We identified FRC as strong source of nitric oxide (NO) thereby directly dampening T cell expansion as well as reducing the T cell priming capacity of DC. The expression of inducible nitric oxide synthase (iNOS) was up-regulated in a subset of FRC by both DC-signals as well as interferon-γ produced by primed CD8+ T cells. Importantly, iNOS expression was induced during viral infection *in vivo* in both LN FRC and DC. As a consequence, the primary T cell response was found to be exaggerated in *Inos* ^−/−^ mice. Our findings highlight that in addition to their established positive roles in T cell responses FRC and DC cooperate in a negative feedback loop to attenuate T cell expansion during acute inflammation.

## Introduction

Adaptive immune responses are initiated most efficiently within secondary lymphoid organs (SLO), such as the spleen or lymph nodes (LN), where pathogens (or foreign antigens) are filtered from body fluids and presented to recirculating T cells. Typically, dendritic cells (DC) residing in peripheral tissues capture foreign antigens and danger signals inducing their maturation, including up-regulation of the chemokine receptor CCR7 allowing DC to migrate via lymphatic vessels into the paracortex (T zone) of the draining SLO. There they present antigen-derived peptides in the context of surface MHC molecules to thousands of naïve recirculating T cells. Only the rare antigen-specific T cells become activated, start secreting cytokines and undergo multiple rounds of cell division. Finally, they differentiate into effector cells and leave the LN as a large cohort that migrates specifically to the site of inflammation [1], [2], [3], [4].

Both in mice and humans, the microenvironment where T cells encounter DC is spanned by a 3-dimensional (3D) network of T zone fibroblastic reticular cells (T-FRC or TRC) known to produce the extracellular matrix scaffold, including microvessels called conduits [2], [4], [5], [6], [7], [8], [9]. More recently it has become clear that TRC are not only cells providing a 3D microenvironment but play an active role in adaptive immunity. They physically guide lymphocytes during their several hours-long migration across the T zone by forming a ‘road system’ [2]. TRC also actively recruit CCR7 expressing T cells and DC into the T zone by constitutively secreting CCL19 and CCL21 [9], [10], [11]. These chemokines not only retain T cells in the T zone but also promote their motility [12]. Furthermore, incoming and resident DC adhere to TRC as well as their associated matrix structures [2], [4], [8]. Finally, TRC are the major constitutive source of IL-7 in LN and access to LN TRC is critical for naïve T cell survival [9], [13].

As the processes of selection, amplification and differentiation of antigen-specific T cells all take place within the TRC environment, it raises the possibility that TRC positively influence these steps. Several lines of evidence support this hypothesis: First, the TRC network appears to increase the frequency of DC-T cell encounters leading to a faster selection of antigen-specific T cells whose frequency is estimated to be around 1 out of 100'000 T cells for a given protein antigen. Both physical and chemical guidance cues provided by TRC are thought to contribute to this effect [2], [4]. Second, the homeostatic chemokines CCL19 and CCL21 act as co-stimulatory signals for T cell activation and proliferation *in vitro* [14], [15]. These chemokines also increase DC maturation and function (reviewed in [16]). Third, IL-7 enhances T cell responses to viral infections *in vivo* [17], [18]. Together, these observations have strengthened the notion that TRC help in the induction of T cell responses by accelerating T cell priming and expansion.

However, recent reports have suggested that TRC may also negatively regulate T cell responses. TRC were shown to express the inhibitory programmed death ligand 1 (PD-L1) thereby reducing CD8 T cell mediated pathology [19]. TRC also express self-antigens in the context of MHC class I thereby promoting CD8^+^ T cell tolerance [20], [21] (reviewed in [22], [23]). In addition, stromal cells isolated from neonatal or adult spleen were shown to induce over 1–2 weeks the development of DC that inhibit T cell proliferation *in vitro*. The spleen contains many stromal cell subsets and the precise identity of the cells used as well as their localization relative to DC and T cells has remained unclear [24], [25]. Together, these observations indicate that lymphoid tissue stromal cells may also inhibit T cell responses.

Currently, the exact role of LN TRC in T cell activation and differentiation is not known. This is in part due to the difficulty of isolating sufficient numbers of TRC for *in vitro* experiments and the lack of appropriate tools to investigate TRC *in vivo*. Here we used a combination of *in vitro* and *in vivo* approaches to study the effect of TRC on CD8^+^ T cell priming by antigen-pulsed DC. We demonstrate that TRC diminish T cell expansion by releasing NO. They share this property with a subset of DC. We show that NO production by TRC and DC is strongly dependent on cytokines from activated T cells suggesting a negative feedback loop once T cell priming has started. Our *in vivo* findings using *Inos* ^−/−^ mice indicate that TRC and DC limit the speed of T cell expansion possibly to ensure organ functionality during the early phase of acute lymph node swelling.

## Results

### Lymph node TRC dampen T cell expansion

To dissect the role of TRC in T cell activation and differentiation, we initially adopted a reductionist approach: T cells were co-cultured with antigen-pulsed bone-marrow derived DC (BM-DC), either in the presence or absence of TRC. Given the difficulties in isolating pure TRC populations, several independent TRC cell lines were established from peripheral lymph nodes (pLN = pool of inguinal, axillary and brachial LN) of C57BL/6 wild-type (WT) mice (referred to as ‘pLN1’ and ‘pLN2’) or GFP-expressing mice (‘GFP-pLN’). All these lines showed typical fibroblastic morphology and a uniform surface marker expression pattern comparable to *ex vivo* isolated TRC (Fig. S1 and data not shown). In contrast to *ex vivo* TRC [9] cell lines expressed only low levels of *Ccl19*, *Ccl21* and *Il7* transcripts. To circumvent this caveat, initial experiments included exogenously added CCL19, CCL21 and IL-7 protein with no difference in the outcome (data not shown).

**Figure 1:**
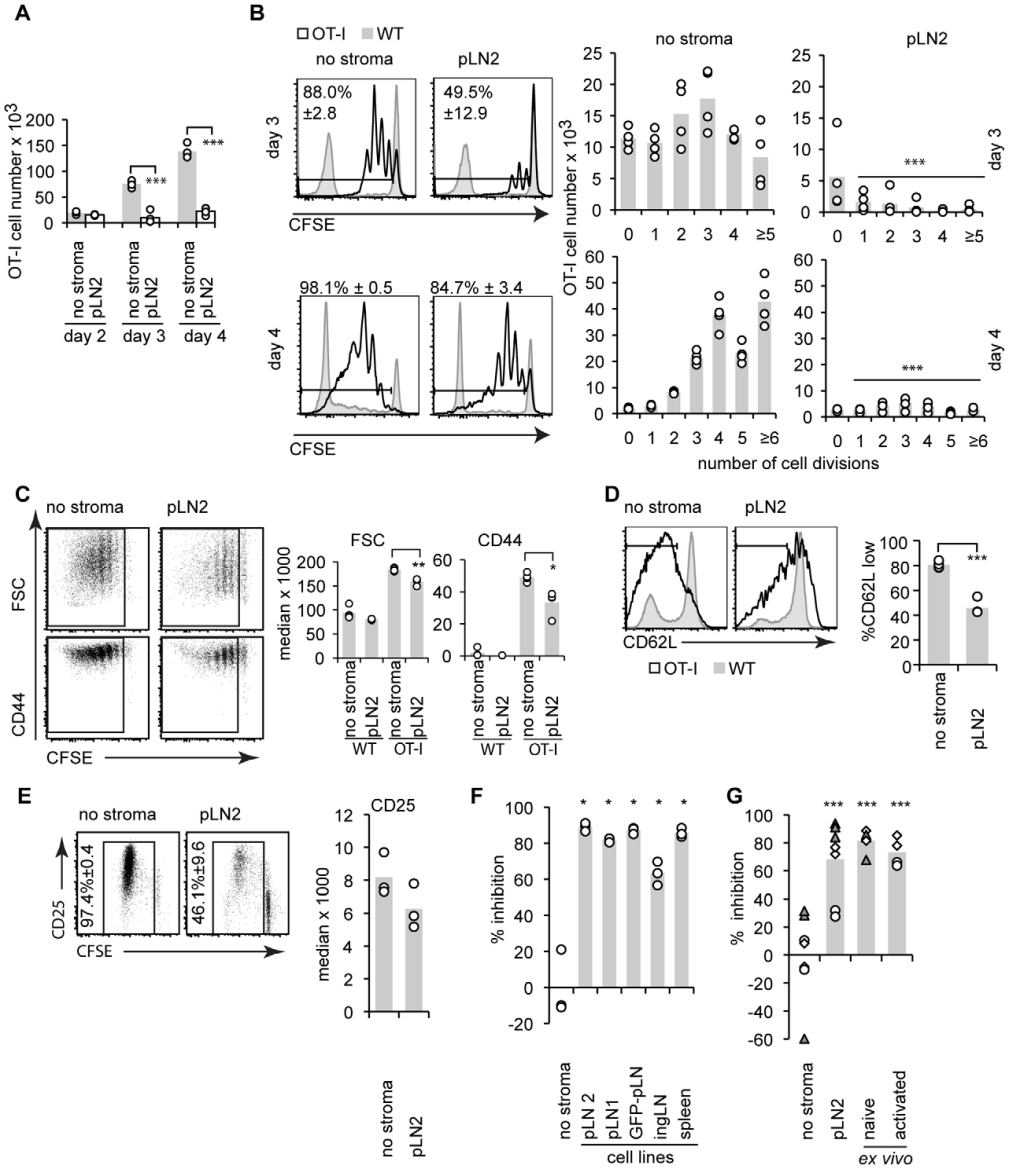
TRC dampen the expansion of antigen-specific CD8^+^ T cells. Flow cytometric analysis of T cell activation: CFSE-labeled OT-I T cells were mixed in a ratio of 1∶50 with WT T cells (50% CFSE-labeled) and cultured with LPS-activated and OVA-peptide pulsed BM-DC. The assay was performed without stromal cells (‘no stroma’) or in the presence of TRC from distinct sources (1∶1∶200 ratio of TRC to BM-DC, and to T cells). (**A**) Total OT-I numbers on day 2, 3 and 4 in absence or presence of the pLN2 TRC line. (**B**) Proliferation of OT-I (black) or polyclonal T cells (gray) as assessed by CFSE dilution on day 3 or 4 with or without pLN2. Histograms (left) show the percentage (± standard deviation) of divided OT-I cells and bar graphs the absolute OT-I cell numbers per CFSE dilution peak (right). (**C**) Dot plots (left) show CFSE dilution versus cell size (FSC) or CD44 expression of OT-I T cells after 3 days. Bar graphs (right) indicate the median FSC values or fluorescence intensity. (**D**) Histograms show CD62L expression and bar graphs the percentage of CD62L^low^ OT-I T cells. (**E**) As c, but showing CD25 expression on OT-I T cells after 4 days of co-culture with or without pLN2. (**F,G**) Bar graphs show the percentage inhibition of OT-I T cell proliferation as based on the proliferating OT-I cell numbers after 3 days of co-culture with different TRC lines (**F**) or *ex vivo* isolated and enriched TRC (**G**). The latter are derived from pLN of unmanipulated B6 mice or from mice s.c. injected with NP-CGG/Montanide 3 days prior to the TRC harvest (‘activated TRC’). Different symbols indicate different experiments. Data are representative of >10 (**A–D**), 3 (**E,G**), 2–3 (**F**) experiments; different symbols indicate different experiments. * *P*<0.05, ** *P*<0.01, *** *P*<0.001, *P*-values relate to ‘no stroma’ controls.

**Figure 2:**
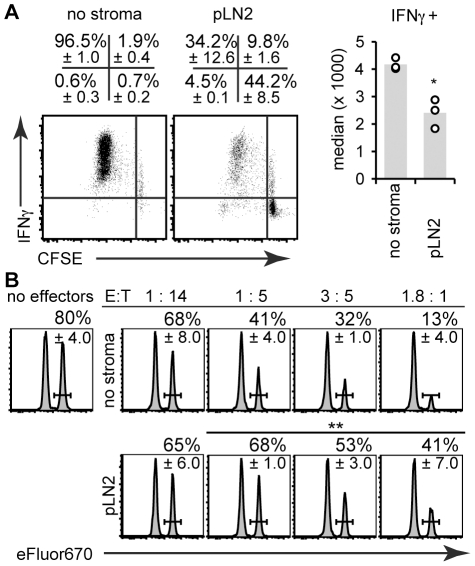
CD8^+^ T cells primed in presence of TRC still produce IFNγ and kill target cells. The T cell activation assay (see legend of Fig. 1) was performed without stroma (‘no stroma’) or in presence of the pLN2 TRC line for 4 days followed by flow cytometric analysis of OT-I T cell effector function. (**A**) Dot plots (left) show intracellular IFN**γ** deposition versus CFSE dilution in OT-I T cells after *in vitro* re-stimulation with 1 µM SIINFEKL peptide in presence of brefeldin A. The right panel shows the median fluorescence intensity of IFN**γ** expression in OT-I T cells. (**B**) The cytotoxic capacity of OT-I T cells was assessed by co-incubating OT-I T cells ( = effector cells, E) from the T cell activation assay in the indicated E∶T ratios with SIINFEKL-pulsed-eFluor670^high^-labeled splenocytes ( = target cells, T) mixed 1∶1 with unpulsed-eFluor670^low^-labeled splenocytes ( = internal control). After overnight culture the percentage of eFluor670^high^ versus eFluor670^low^ splenocytes was analyzed and plotted as histograms. Indicated as percentage is the survival index for the target cells, as based on the ratio of peptide-pulsed eFluor670^high^ relative to unpulsed eFluor670^low^ cells (± standard deviation). (**A, B**): n = 3, representative of 3 independent experiments. * *P*<0.05, ** *P*<0.01, *** *P*<0.001, *P*-values are relative to the corresponding ‘no stroma’ control.

To study T cell priming CD45.1^+^ congenic ovalbumin (OVA)-specific OT-I T cell receptor (TCR) transgenic CD8^+^ T cells were labeled with the proliferation dye carboxyfluorescein succinimidyl ester (CFSE), mixed with unspecific WT T cells (CD45.2^+^) in a ratio of 1∶50, and cultured together with antigen-pulsed BM-DC on top of an adherent layer of the TRC line. TRC were previously irradiated to limit their proliferation and nutrient consumption. Surprisingly, the total OT-I cell number was strongly decreased in presence of the TRC line pLN2 (Fig. 1A). Using CFSE dilution to measure T cell proliferation, both the percentage and number of dividing OT-I T cells were strongly reduced in the presence of pLN2 (Fig. 1B). The increase in cell size (FSC) and CD44 expression (Fig. 1C) as well as the loss of CD62L expression (Fig. 1D) occurred in presence of TRC but to a reduced extent. The co-cultures were supplemented with IL-7 and IL-2, so a lack of known pro-survival factors for naïve and activated T cells is unlikely to be the cause. In line with that, the number of naïve, undivided OT-I T cells was not affected by the TRC presence, nor was the up-regulation of the high-affinity receptor chain for IL-2, CD25, on dividing T cells (Fig. 1E). Importantly, several other fibroblast lines established independently from LN and spleen [26] not only shared the same surface phenotype (Fig. S1) but also the inhibitory effect on T cell expansion with a reduction in proliferating OT-I T cell numbers of 60–90% (Fig. 1F). Importantly, primary TRC isolated from naïve pLN limited T cell expansion at least as strongly as TRC lines (Fig. 1G). Even TRC isolated from pLN of mice immunized 3 days earlier with NP-CGG in Montanide adjuvant maintained these inhibitory properties (Fig. 1G). Next, we examined the effect of TRC on CD8^+^ T cell differentiation. OT-I T cells primed in presence of TRC expressed intracellular interferon gamma (IFN**γ**) protein (Fig. 2A) and killed target cells (Fig. 2B), although with markedly reduced efficiency (Fig. 2B). Together these results demonstrate that the presence of TRC during T cell activation diminishes the expansion or survival of CD8^+^ T cells and to a lesser extent their differentiation into effector cells.

### Fibroblasts from non-lymphoid organs also attenuate T cell proliferation

**Figure 3:**
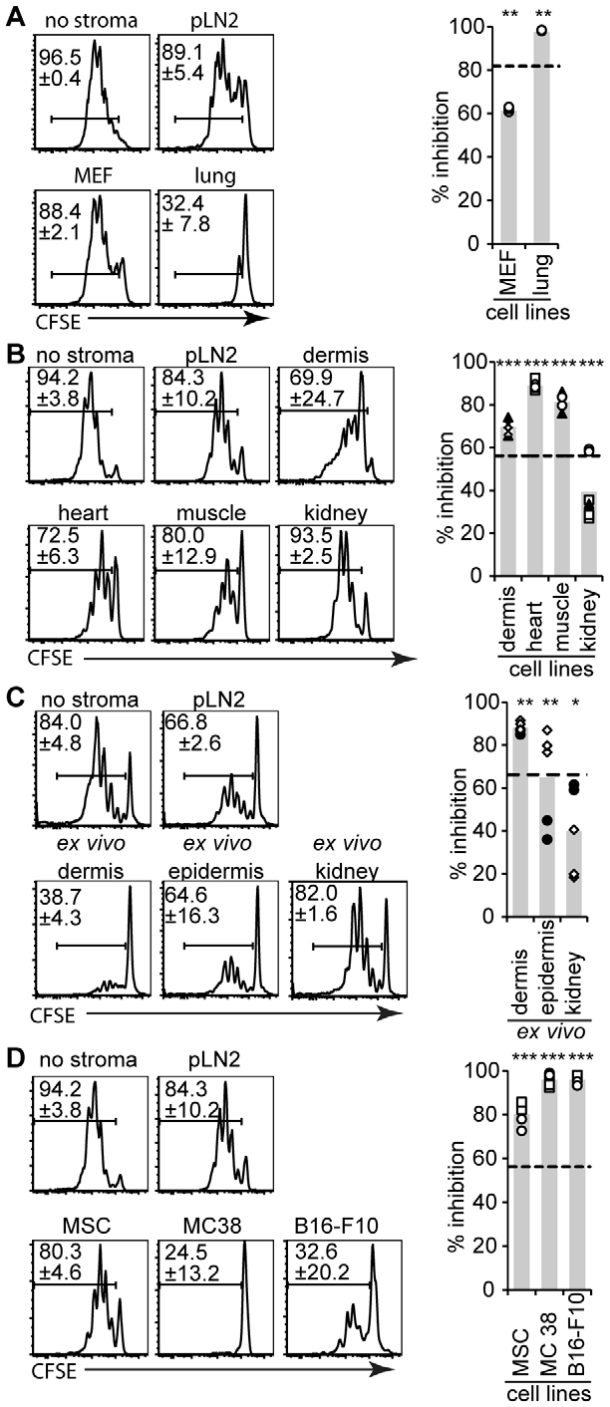
Stromal cells from various non-lymphoid organs decrease antigen-specific T cell proliferation. The T cell activation assay (see legend of Fig. 1) was performed in presence of either stromal cell lines (**A,B,D**) or *ex vivo* isolated stromal cells (**C**). OT-I T cells co-cultured for 3 days were analyzed for CFSE dilution using flow cytometry. Representative histograms of CFSE dilution (left panel) and the number of proliferating OT-I cells as percentage inhibition are presented (right panel, as in Fig. 1). Shown are assays with a (**A**) mouse embryonic fibroblast line (MEF) and lung fibroblast line, (**B**) lines derived from different non-lymphoid organs (dermis, heart, femoral muscle, kidney), (**C**) *ex vivo* isolated adherent cells from the dermis, epidermis or kidney, (**D**) Mesenchymal stem/stromal cell (MSC) line and the tumor cell lines B16-F10 (melanoma) and MC-38 (colon carcinoma). (**A–D**) n≥3, representative for ≥2 experiments. Different symbols indicate different experiments. * *P*<0.05, ** *P*<0.01, *** *P*<0.001, if not indicated otherwise p-values are relative to ‘no stroma’. The dotted line indicates the inhibition observed by pLN2 cells within the same experiment.

It has been reported that murine and human fibroblasts can have anti-proliferative effects on activated T cells, similar to mesenchymal stem cells (MSC) and certain tumor lines [27], [28], [29], [30], [31]. Therefore we tested in our system the inhibitory potential of several fibroblastic cell lines established *de novo* from different non-lymphoid organs, as well as their *ex vivo* equivalents (CD45^−^CD35^−^CD31^−^EpCAM^−^gp38^+^; see Fig. S2), and compared them to our pLN2 line. In addition, epithelial-like cells from the epidermis and kidney, a MSC line and two tumor cell lines (MC38 colon carcinoma and B16-F10 melanoma) were tested. All cell lines inhibited OT-1 T cell proliferation to an extent comparable with pLN2 (Fig. 3 A–D), highlighting that inhibition of T cell expansion is a property common to many cell types, including fibroblasts from various non-lymphoid organs.

### TRC directly inhibit T cell proliferation and render DC less stimulatory

**Figure 4:**
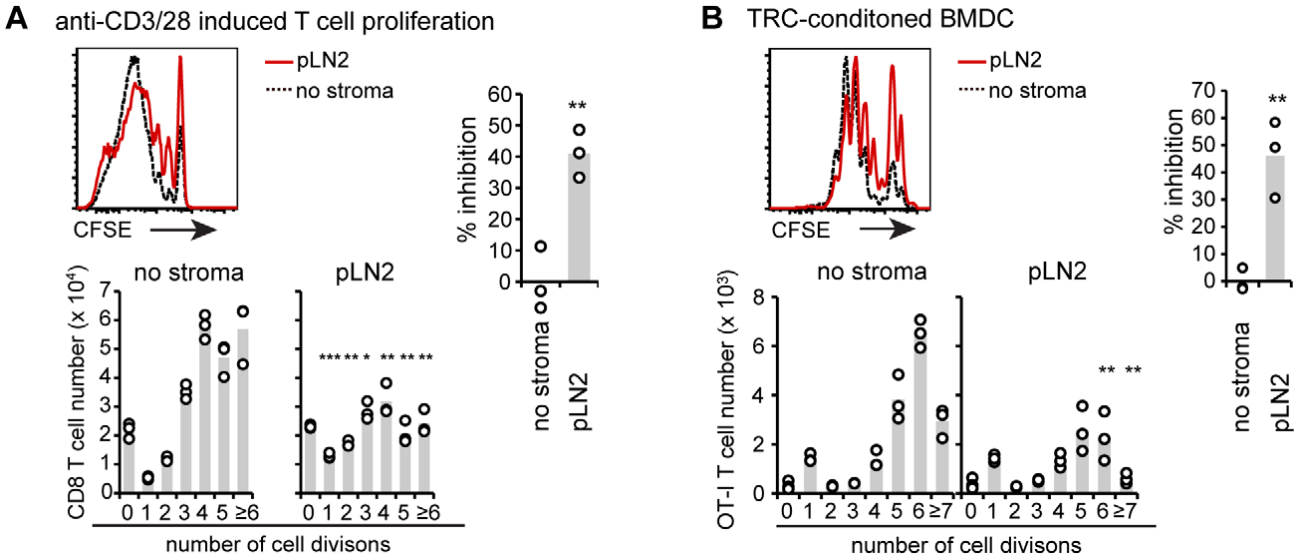
TRC dampen T cell proliferation by modifying both T cells as well as BM-DC. (**A**) Flow cytometric analysis of T cell proliferation induced by anti-CD3/CD28 beads as based on CFSE dilution after 3 days of culture. WT T cells were cultured with beads and in presence of the pLN2 TRC line (T cell to TRC ratio of 100∶1) or their absence (‘no stroma’). Representative histograms (top) show the percentage (± standard deviation) of divided CD8^+^ T cells and bar graphs (bottom) the absolute CD8^+^ T cell numbers per division. The bar graph (right) shows percentage of inhibition (as in Fig. 1). Similar data were obtained for CD4^+^ T cells suggesting TRC may also suppress their expansion (data not shown). (**B**) Flow cytometric analysis of cell proliferation induced by TRC-conditioned BM-DC. LPS-activated and SIINFEKL-pulsed BM-DC were co-cultured with pLN2 TRC at a ratio of 1∶1, or cultured alone. After overnight culture TRC-conditioned-BM-DC were re-isolated by MACS, counted and subsequently co-cultured for 3 days with CFSE labeled OT-I cells mixed 1∶50 with unspecific WT T cells (of which 50% are CFSE labeled). Representative histograms (top) show the percentage (± standard deviation) of divided OT-I cells and bar graphs (bottom) the absolute cell numbers per CFSE dilution peak. Percentage of inhibition (as in Fig. 1) is shown in the bar graph on the right. (**A,B**) n = 3, (**A**) representative for ≥3 experiments, (**B**) representative for 2 experiments. * *P*<0.05, ** *P*<0.01, *** *P*<0.001, if not indicated otherwise *P*-values are relative to ‘no stroma’.

MSC have been shown to inhibit T cell proliferation by intracellular expression of enzymes such as indoleamine 2,3-dioxygenase (IDO) and arginase-1, surface expression of PD-L1, or by secretion of molecules like NO, prostaglandin E_2_ (PGE_2_), IL-10 and TGFβ(reviewed in [30], [31]). The proposed pathways included direct inhibition of T cell proliferation or conversion of DC to a non-stimulatory or suppressive phenotype (reviewed in [31], [32]). To examine whether TRC can directly interfere with T cell proliferation pLN2 were co-cultured with anti-CD3/CD28 bead-stimulated T cells. Clearly, TRC diminished the number of dividing T cells but the inhibitory effect was only half of what we had observed in the BM-DC-induced T cell activation assay (Fig. 4A). Next, potential effects of TRC on the stimulatory capacity of antigen-pulsed BM-DC were investigated by co-culturing first pLN2 with BM-DC before incubating these ‘TRC-conditioned’ BM-DC alone with T cells. The capacity to stimulate T cell proliferation was reduced by half in conditioned relative to unconditioned BM-DC (Fig. 4B). Together these results indicate that TRC limit T cell expansion in two additive ways, by making DC less immunogenic and by directly inhibiting T cell proliferation or survival.

### TRC limit T cell expansion by producing NO

**Figure 5:**
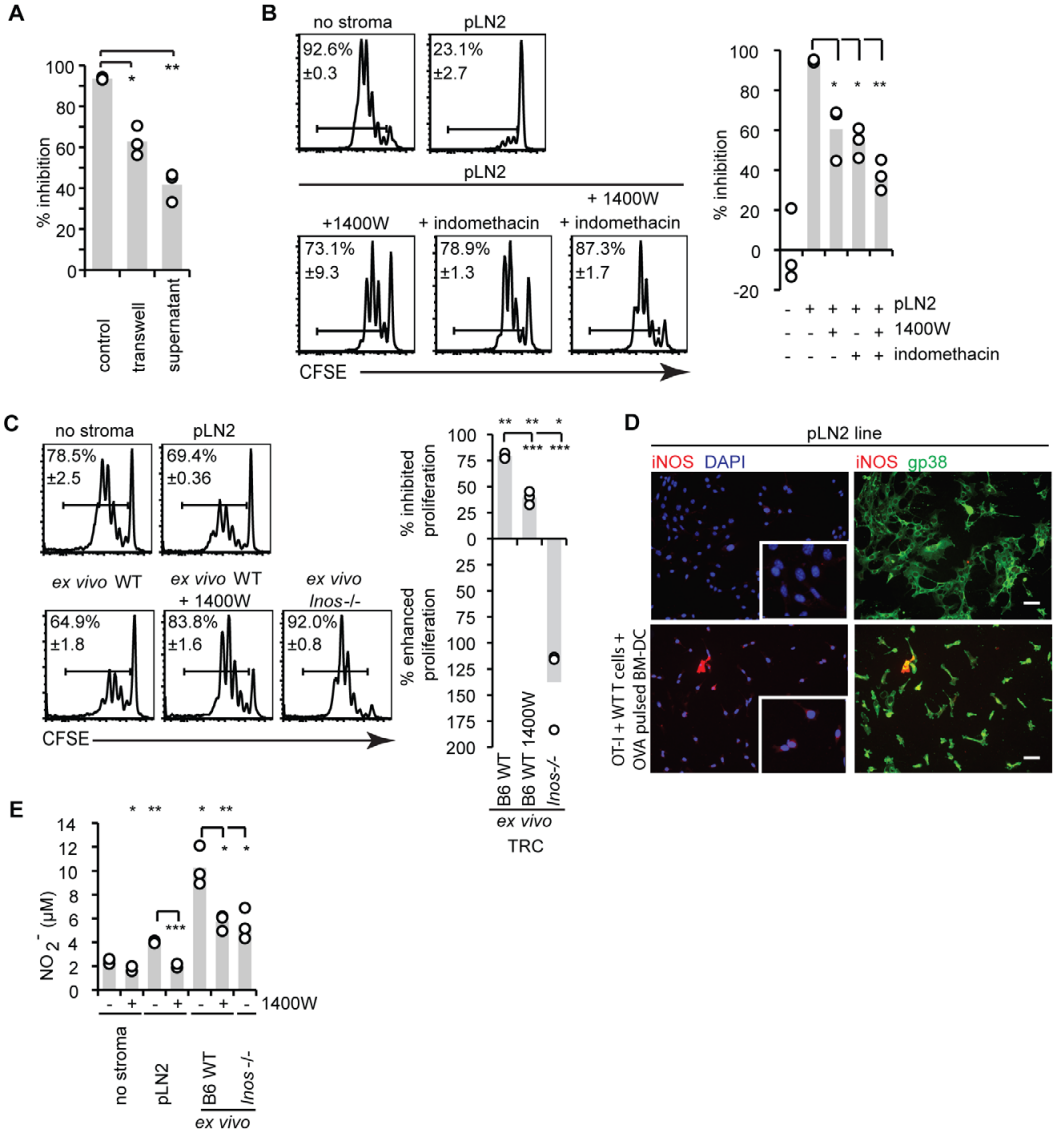
Suppression by TRC is mediated via NO and COX-1/2 dependant factors. (**A,B,C**) Flow cytometric analysis of the T cell activation assay (as described in Fig. 1): (**A**) pLN2-TRC in the assay were present in the same well (‘normal’), separated by a permeable membrane (transwell) or 30% pLN2-TRC-conditioned-supernatant taken after 72 h from sub-confluent irradiated cultures was added to the cultures. The bar graph shows percentage of inhibition. (**B**) Pharmacological inhibitors against iNOS (1400W, 1 µM) and/or Cox-1/2 (indomethacin, 10 µM) were added to the co-cultures after optimization of their concentrations (tested range for 1400W: 1–10 µM; for indomethacin: 1–10 µM). The middle panel shows the percentage of divided OT-I cells (± standard deviation) and the bar graph (right) shows the percentage of inhibition. (**C**) T cell activation assay was carried out in the presence of *ex vivo* isolated enriched TRC from WT or *Inos* ^−/−^ mice along with the indicated inhibitors. The right panel shows the percentage of divided OT-I cells (± standard deviation) and the bar graph (middle) shows the percentage of inhibition. (**D**) Immunohistochemical analysis of iNOS expression in pLN2 TRC lines. pLN2 cells were cultured alone (top) or in presence of OT-I and WT T cells together with LPS activated, SIINFEKL-pulsed BM-DC (ratios as in Fig. 1) (bottom). Insets of higher resolution images are shown to indicate the generally increased iNOS expression level in pLN2 co-cultured with BM-DC, antigen and T cells. (**E**) Bar graph showing NO_2_^−^ levels in the co-culture supernatant from the T cell activation assay shown in (b,c) as determined using a Griess assay. (**A,B,C,E**) n = 3, (**D**) n = 3–4, (**A,C**) representative for 2, (**B**) representative for ≥3 experiments, (**D**) 1 experiment; * *P*<0.05, ** *P*<0.01, *** *P*<0.001, if not indicated otherwise *P*-values are relative ‘no stroma’.

To gain insight into the nature of the TRC-derived inhibitory factor T cells and BM-DC were either separated from the pLN2 by a permeable-membrane (transwell) or simply incubated with supernatant (SN) from pLN2. Both settings led to a decrease in T cell expansion, but this was smaller than in co-cultures where all the three cell types are intermingled (Fig. 5A). These results suggest a role for either a soluble factor which acts only efficiently at short distance, or for at least two inhibitory factors, one being soluble and the other being cell-cell contact dependent. It also argues against a role for TRC in presenting antigen to T cells and thereby inhibiting T cell proliferation.

To identify the molecules responsible for suppressing T cell expansion in our assay several candidates or their synthesis pathway were blocked, including PD-L1, TGFβ, IL-10, IDO and arginase-1 but none of them neutralized the TRC-mediated inhibition (Fig. S3). However, blocking the enzymatic activity of iNOS (NOS2) or cyclooxygenase (Cox) −1 and −2 partially rescued T cell proliferation with the effects of the two pharmacological inhibitors used being partially additive (Fig. 5B). This result demonstrates that iNOS-dependent NO and Cox-1/2 dependent factors are responsible for most of the inhibitory effect observed. We focused our attention on iNOS as transcripts for *Inos* but not *Cox2* were enhanced in stimulated TRC (as shown later). Strikingly, *Inos* ^−/−^ TRC had lost all suppressive activity and showed even an enhancement of T cell proliferation, in contrast to the inhibitor 1400W that partially abolished the suppressive effect of *ex vivo* WT TRC (Fig. 5C), reminiscent of the effects seen for the pLN2 line (Fig. 5B). To show directly the expression of iNOS protein in TRC and assess the frequency of iNOS-expressing cells, antibodies to iNOS were used in immunofluorescence microscopy. Surprisingly, iNOS protein could only be detected in pLN2 co-cultures with BM-DC and activated T cells. However, iNOS was only detected in a small fraction of gp38^+^ TRC (Fig. 5D). Occasionally, iNOS protein staining was observed in BM-DC-like cells (gp38^−^) but never in lymphocytes (not shown). These experiments establish that TRC are a source of iNOS that inhibits T cell proliferation. They also demonstrate that the inhibitory activity of TRC on T cell expansion is not due to metabolic effects of co-culture.

As a readout of iNOS activity, we measured extracellular nitrite levels in the SN. In absence of TRC the SN of T cells activated by BM-DC contained very low levels of nitrite. Presence of pLN2 or *ex vivo* TRC in this assay increased the NO_2_^−^ concentration 2- and 5-fold, respectively (Fig. 5E), further strengthening the notion that NO production by TRC leads to inhibition of T cell proliferation or survival. In the case of *ex vivo* TRC approximately half of the nitrite was due to iNOS activity in TRC as suggested by the use of 1400W or *Inos* ^−/−^ TRC. They also suggest a contribution of NO production by one of the two other NOS isoforms which are insensitive to the concentrations of 1400W used and possibly expressed in TRC or in the few endothelial cells found within the adherent cell fraction. As the levels of iNOS protein correlate better with NO-mediated inhibition of T cell expansion than those of extracellular nitrite, the subsequent analysis was focused on the factors inducing iNOS expression in TRC.

### IFNγ and other T cell derived cytokines induce iNOS protein expression in TRC

**Figure 6:**
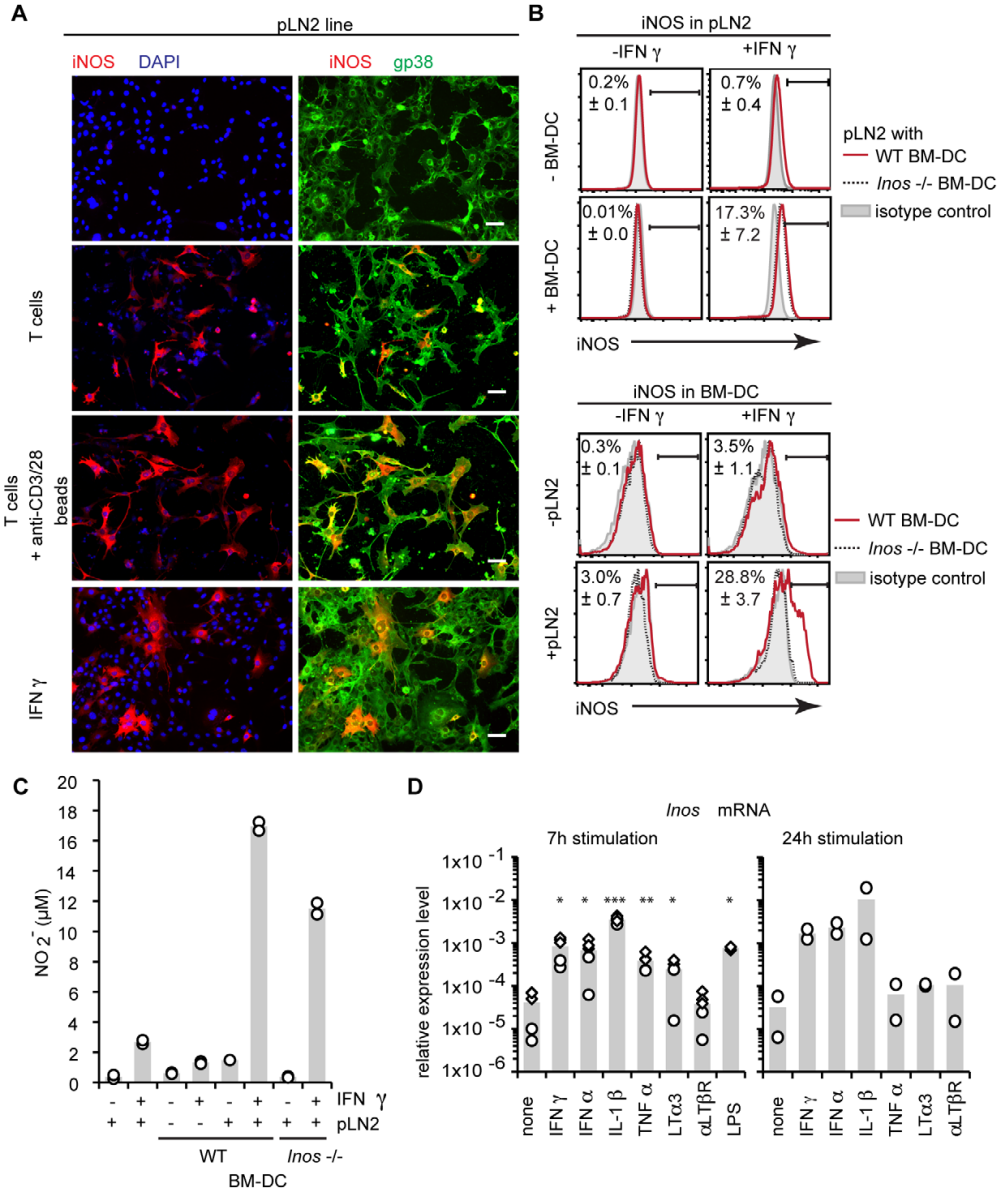
iNOS expression in TRC can be induced by IFNγ. (**A**) Immunohistochemical analysis of iNOS expression in pLN2 TRC lines. pLN2 were cultured for 2 days either alone (1st row), in the presence of WT T cells without (2nd row), with anti-CD3/28 beads (3rd row), or in the in the presence of recombinant IFN**γ** bottom row. The first column shows iNOS expression in red and nuclei stained with DAPI in blue, the second column shows iNOS in red and gp38^+^ TRC in green. Tiny nuclei are from T cells, larger nuclei from BM-DC and pLN2. Scale bar: 50 µm. (**B**) Flow cytometric analysis of intracellular iNOS expression in pLN2 TRC or BM-DC, cultured for 2 days either alone or together and with or without recombinant IFN**γ**. The histograms show the % of iNOS-expressing TRC (gp38^+^CD45^−^CD31^−^; upper panel) or BM-DC (CD45^+^; lower panel) (± standard deviation). (**C**) NO_2_^−^ concentration in the supernatant from the co-cultures shown in (b). (**D**) Quantitative RT-PCR analysis for *Inos* mRNA of pLN2 cells stimulated with various cytokines or agonistic anti-LTβR (αLTβR) antibody for 7 h or 24 h. The expression levels relative to two housekeeping genes are shown. Different symbols indicate different experiments. (**A,D**) n = 3–5, representative for 2 experiments. (**B,C**) n = 2, 1 experiment; * *P*<0.05, ** *P*<0.01, *** *P*<0.001; if not indicated otherwise *P*-values are relative to ‘no stroma’.

To look at the role of T cells in inducing iNOS protein in TRC, pLN2 were co-cultured with T cells and fixed cells analyzed for intracellular iNOS protein by immunohistology. Non-activated T cells induced iNOS in only a few TRC whereas T cells activated with anti-CD3/CD28 beads up-regulated iNOS in a large proportion of TRC (Fig. 6A). This suggests that most TRC have the potential to express iNOS upon activation but typically only a small proportion shows detectable protein. IFN**γ** is a T cell-derived cytokine known to induce iNOS expression in various cell types [33], [34], [35], [36]. Indeed, adding recombinant IFN**γ** was sufficient to induce iNOS expression in a fraction of pLN2 (Fig. 6A). Comparable results were obtained with *ex vivo* LN cells when the adherent cells enriched for gp38^+^CD31^−^ TRC were used in presence of naïve or activated T cells or IFN**γ** (Fig. S4A). Consistent with these data a significant increase in nitrite was observed in the SN of TRC lines or *ex vivo* TRC upon IFN**γ** stimulation or upon addition of T cells with or without anti-CD3/28 activation (Fig. S4B). To obtain a more quantitative assessment of iNOS-expressing TRC, iNOS-expressing cells were measured using intracellular staining and flow cytometry. This analysis showed that while IFN**γ** induced few TRC to become iNOS-positive, there was a strong synergistic effect when both IFN**γ** and BM-DC were present in the co-culture with up to 25% TRC being positive (Fig. 6B). Given that BM-DC were occasionally observed to express iNOS in the co-cultures, we assessed the influence of IFN**γ** and TRC on the ability of BM-DC to express iNOS. Interestingly, both IFN**γ** and TRC induced iNOS protein expression in around 3% of BM-DC, but having both IFN**γ** and TRC in the co-culture led to a strong synergistic iNOS expression in up to 30% of BM-DC (Fig. 6B). This synergy was reflected in the almost 10-fold increase in extracellular nitrite levels detectable in the SN (Fig. 6C). Interestingly, more than 50% of the nitrite seemed to be due to iNOS expression in TRC rather than BM-DC as assessed using *Inos* ^−/−^ BM-DC. At present, a contribution by the two other NOS isoforms to nitrite production can not be excluded.

Next we analyzed the speed of iNOS induction in pLN2 by looking at transcript levels. Already 7 h after IFN**γ** stimulation *Inos* mRNA was induced 10-fold and further increased after 24 h indicating direct transcriptional regulation of the *Inos* promoter (Fig. 6D). Interestingly, several pro-inflammatory cytokines (IFNα, TNFα, LTα3) or LPS showed a similarly rapid and strong induction of *Inos* transcripts while their maintenance at 24 h differed. In summary, these results demonstrate that a subset of both TRC and BM-DC can be triggered to express *Inos* transcripts and proteins leading to the release of NO. Various pro-inflammatory signals can serve as triggers in TRC, including signals derived from newly primed T cells suggesting the possibility of a negative feedback loop leading to inhibition of T cell expansion in our *in vitro* co-culture assay.

### *Inos* deficiency leads to exaggerated CD8^+^ T cell responses *in vivo*

**Figure 7:**
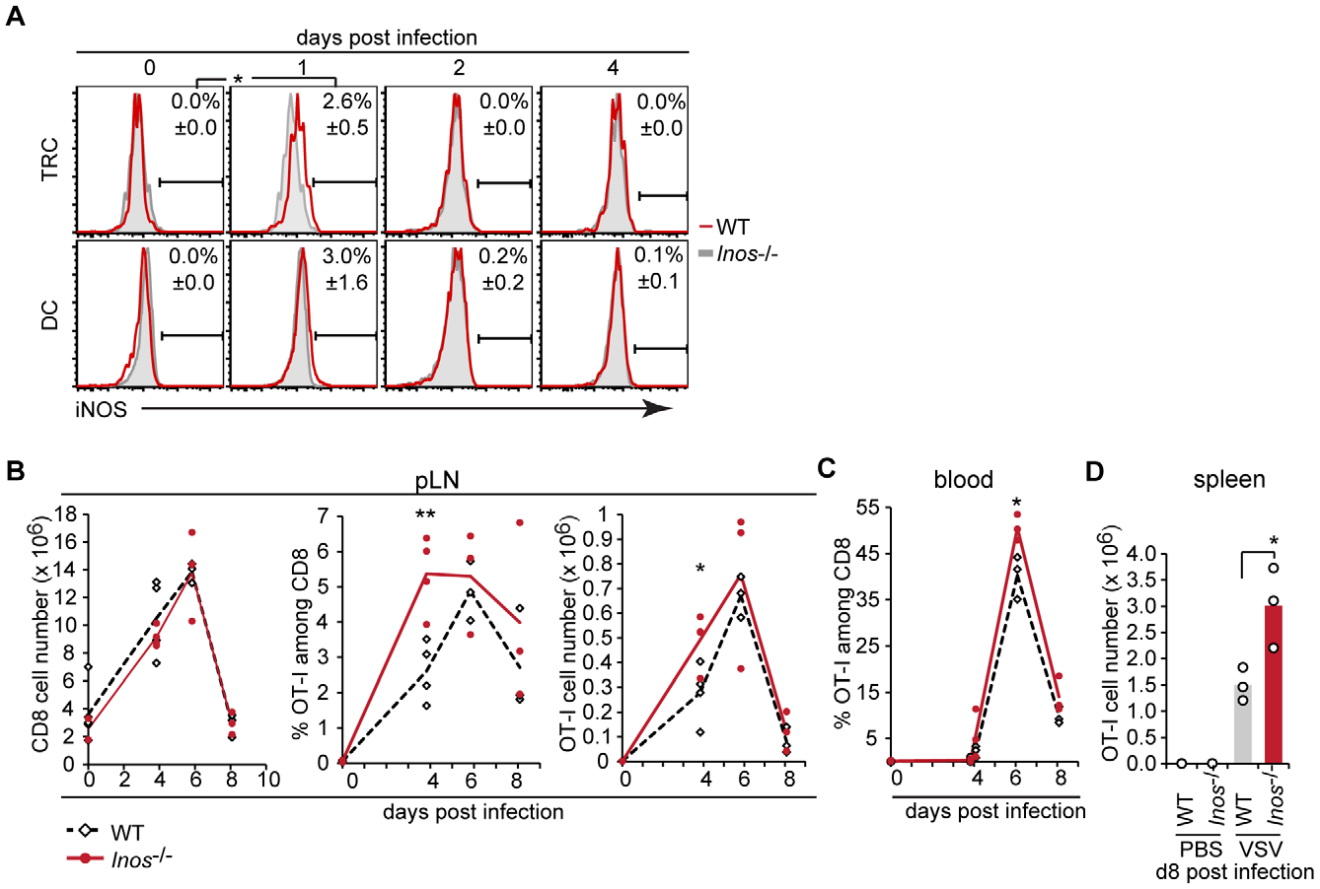
Lack of iNOS *in vivo* leads to increased an primary T cell response after viral infection. (**A–D**) WT or *Inos* ^−/−^ mice were retro-orbitally grafted with 100'000 splenocytes from OT-I transgenic mice one day prior to subcutaneous infection with VSV-OVA. Draining pLN, blood or spleen from infected mice were harvested at the indicated time points after infection and cell suspensions generated by using collagenase digestion (**A**) or by mechanical homogenization (**B, D**). (**A**) Intracellular iNOS protein expression in TRC (gp38^+^CD45^−^CD31^−^CD35^−^) or DC (CD11c^+^MHCII^+^CD45^+^) as analyzed by intracellular flow cytometry. Representative histograms show the percentage (± standard deviation) of iNOS expressing TRC (top) and DC (bottom) in WT versus *Inos* ^−/−^ mice. (**B**) The graph on the left side shows the total CD8^+^ T cell numbers, the graph in the middle the percentage of OT-I T cells among the CD8^+^ T cells and the graph on the right side the total OT-I number in pLN (pool of 6 draining LN). No significant difference was observed in total LN cell numbers between immunized WT and *Inos* ^−/−^ mice (not shown). (**C**) Shows the percentage of OT-I T cells among the CD8^+^ T cells in the blood, (**D**) shows the total number of OT-I T cells in the spleen. (**A–D**) n≥3, 1 out of 2 independent experiments is shown. * *P*<0.05, ** *P*<0.01, *** *P*<0.001.

To characterize the expression of iNOS during immune response *in vivo*, mice adoptively transferred with OT-I T cells were immunized with OVA-expressing vesicular stomatitis virus (VSV-OVA) and draining LN analyzed. By pressing LN across a 40 µm mesh a soluble fraction containing most hematopoietic cells was obtained as well as a non-soluble stromal cell fraction, including TRC and part of the DC [9], [10]. Interestingly, one day after immunization *Inos* transcripts were induced in both fractions and dropped back to pre-immunization levels on day 2 and 4 (Fig. S5A). These levels were always 5–10-fold higher in the fraction enriched in stromal relative to hematopoietic cells. To identify more precisely the cell type and frequency of iNOS expressing cells in the draining LN intracellular iNOS protein expression was analyzed by flow cytometry along with lineage markers. A subset of at least 2.6% TRC were identified as major iNOS source appearing on day 1 and being again undetectable on day 2 and 4 post infection (Fig. 7A). DC showed a weak induction of iNOS expression on day 1 without reaching statistical significance relative to day 0. Few macrophages (CD11b^+^ CD11c^−^) were also found to express iNOS protein (data not shown). This result emphasizes the inducible and transient activation of iNOS both at the mRNA and protein level, consistent with pro-inflammatory molecules inducing it. It also strengthens the notion of more than one cell type expressing iNOS within the T zone of draining LN, most notably TRC and DC. Our attempts to identify and localize iNOS protein-expressing cells in situ were not successful (data not shown), consistent with the low iNOS expression detected by flow cytometry. As a next step the impact of *Inos*-deficiency on OVA-specific T cell expansion and survival was measured in the LN draining the site of VSV-OVA injection. While on day 4 after immunization the total CD8^+^ T cell number was similar in both mouse strains the percentage and number of OT-I T cells was 2-fold higher in *Inos* ^−/−^ relative to WT mice (Fig. 7B). On day 6 and 8 after immunization, the difference in OT-I T cell numbers was minimal between the two strains presumably due to many effector cells having left the LN between day 5 and 8. This scenario is supported by the strong increase in OT-I cells in blood between day 4 and 6 as well as by the significantly higher frequency of OT-I cells in blood of *Inos* ^−/−^ relative to WT mice (Fig. 7C). As a consequence 2-fold more effector OT-I T cells accumulated within day 8 spleen in *Inos* ^−/−^ relative to WT mice (Fig. 7D). Interestingly, the differentiation into effector cells occurred efficiently in the absence of iNOS, as based on the analysis of IFN**γ** expression and *in vitro* killing activity of effector CD8^+^ T cells from LN and spleen (Fig. S5 B,C). To address whether iNOS expression is critical in hematopoietic or non-hematopoietic cells bone marrow chimeras were generated. Unfortunately, a cell trapping defect was observed in inflamed LN if the stromal cell compartment was *Inos*-deficient, presumably due to a role of NO in vasodilation in irradiated mice but not straight *Inos* ^−/−^ mice (Fig. S6). Therefore, no conclusions could be drawn from these experiments. In summary, the *in vivo* data indicate that acute inflammation associated with a viral infection leads to the transient expression of NO by TRC and DC found within the LN T zone, which slows down and lowers the antigen-specific T cell expansion but does not seem to impact on the differentiation and migration of effector T cells.

## Discussion

Inflamed LN as well as the TRC network are generally considered as strongly immune-stimulatory for T cell immunity. Surprisingly, we obtained several lines of evidence demonstrating an inhibitory role of TRC in early T cell activation: 1) *In vitro* TRC limit the expansion of CD8^+^ T cells primed by antigen-pulsed BM-DC or anti-CD3/28 beads at TRC-T cell ratio's as low as 1∶100 with T cell effector function being reduced as well. 2) TRC reduce the T cell activation potential of antigen-loaded BM-DC. 3) TRC constitutively express transcripts for Cox-2, the enzyme required for the synthesis of PGE_2_ and related factors [37]. Inhibition of Cox-1/2 markedly reduces this suppressive effect. 4) When exposed to pro-inflammatory cytokines pLN2 and *ex vivo* TRC transiently express iNOS protein leading to NO_2_^−^ synthesis. This correlates with rapid induction of *Inos* transcripts in activated pLN2. Inhibition of iNOS or use of *Inos*-deficient TRC markedly reduces the suppression with this effect being enhanced by a Cox-2-inhibitor. 5) Upon immunization, iNOS protein gets transiently expressed in TRC and DC within the draining LN. 6) Absence of iNOS *in vivo* correlates with an exaggerated primary CD8^+^ T cell response, consistent with the previous observation of an exaggerated memory T cell response in *Inos* ^−/−^ mice [38]. Thus, while TRC may have several ways of enhancing T cell activation, our assays have only revealed attenuating effects.

Attenuating effects of TRC on T cell immunity have been reported recently based on the expression of self-antigen in the context of MHC I as well as PD-L1 expression on TRC leading to modulation of CD8^+^ T cell responses [19], [20], [21]. Here we have used fibroblasts of the LN T zone to show that they are a source of soluble immunomodulators such as NO and COX-1/2 dependent factors strongly inhibiting T cell expansion in response to a foreign antigen, with no role observed for PD-L1. Another study had noted iNOS expression in reticular LN fibroblasts upon infection with *Leishmania major* parasites, but the precise nature and localization of these cells have remained unclear. In addition, NO was in that case shown to be important for the control of the parasite rather than for adaptive immunity [34]. Together these data suggest TRC may help control CD8^+^ T cell responses to both self- and foreign-antigens. Whether they also modulate the deletion of self-reactive T cells still needs to be tested.

Two previous reports have highlighted the capacity of spleen-derived stromal cells in inhibiting T cell activation. A mix of adherent stromal cells, possibly including TRC, was shown to promote development of BM-derived progenitor cells into IL-10^+^ regulatory DC [25] or the conversion of mature BM-DC into NO^+^ regulatory DC [24]. We did not observe consistent changes in the surface phenotype of TRC-conditioned DC, possibly because our co-cultures were less than 16 h allowing only short-term changes. In contrast, the previous reports were based on co-cultures of 1–2 weeks allowing developmental changes. While we have not yet identified the reason for the decreased stimulatory capacity of TRC-conditioned DC it may be due to the rapid induction of iNOS in BM-DC.

Our results are reminiscent of observations with MSC known to modulate many aspects of innate and adaptive immunity, including T cell proliferation [30], [31]. *In vitro* BM-derived MSC were shown to block mitogen- and DC-induced T cell proliferation by many different pathways, including NO and PGE_2_ production [36], [39], [40]. In settings of allografts and autoimmune disease large numbers of injected MSC strongly suppress T cell responses due to their anti-inflammatory properties, and due to their efficient homing to inflammatory sites and lymphoid tissues [30], [31], [41]. Interestingly, mature mesenchymal cells such as fibroblasts from various non-lymphoid tissues also dampen T cell expansion [27], [28], [29], [31] suggesting mesenchymal cells at several maturation stages share this property. Alternatively, MSC and fibroblasts may be hard to distinguish [28]. We propose that LN and spleen TRC have conserved this immunosuppressive property that they share with MSC and fibroblasts from non-lymphoid tissues. The extent of inhibition was highly dependent on the number of TRC being present in the culture (unpublished observation) which may explain the difference in the extent of NO-mediated suppression observed *in vitro* versus *in vivo*. Our preliminary results suggest that this inhibitory effect not only affects CD8^+^ but also CD4^+^ T cells. Besides affecting T cell expansion and possibly apoptosis, TRC presence partially reduced T cell effector differentiation *in vitro* but not *in vivo*. While the reason for this difference is not known, it may be explained by the presence of lower NO concentrations or additional factors found *in vivo* that positively influence CD8^+^ T cell differentiation. Given that TRC and non-lymphoid fibroblasts can be grown easily from many accessible tissues in humans, such as tonsils or skin, they may represent an interesting source of immunomodulatory cells. A very recent study using skin fibroblasts has established their potential in efficiently suppressing the clinical signs of experimental arthritis in mice [29].

Different mechanisms of suppression have been described for MSC: they can either directly inhibit T cell activation or reduce the stimulatory capacity of DC by using several pathways [30], [31]. MSC can produce high amounts of NO upon contact with T cells, which interferes with STAT-5 phosphorylation and therefore IL-2 signaling [42]. MSC-mediated T cell suppression could be partially neutralized using inhibitors of iNOS or Cox-2, or using *Inos* ^−/−^ MSC [36], [40] thus showing clear parallels in MSC- and TRC-mediated T cell mechanisms. In our *in vitro* experiments we have obtained evidence for an additive inhibitory effect of NO and COX-1/2-dependent factors (possibly PGE_2_), but additional factor(s) probably remain to be identified to explain the entire inhibitory effect. Blocking TGFβ, PD-L1, IL-10, arginase-1 and IDO activity did not influence TRC mediated inhibition, however, additional studies will be necessary to fully rule out their contribution. Furthermore, we cannot exclude that the other two NOS isoforms, eNOS and nNOS, are expressed in TRC and contribute to the TRC mediated inhibition.

The expression and activity of iNOS is tightly controlled at several levels with the best-characterized positive regulators being IFN**γ** IFNαβ, TNFα IL-1 and LPS [35]. Indeed, iNOS expression in TRC was induced by all of these pro-inflammatory cytokines confirming and extending previous data using LN fibroblasts, MEF and MSC [33], [34], [35], [36]. Interestingly, only a subset of IFN**γ**-stimulated TRC expressed iNOS, even when a TRC clone was used (data not shown), in line with previous reports on other fibroblast types [33], [34] as well as our observation on BM-DC reported here. Recent results suggest that IFN**γ** is not restricted to immune synapses but more widely accessible throughout the T zone [43] which may explain why TRC obtain this signal in the absence of cognate interaction with primed T cells. Several reports showed that IFN**γ** during T cell priming influences the degree of CD8^+^ T cell contraction (reviewed in [44]). While IFN**γ** signaling within T cells may lead to this outcome [44], our results suggest that IFN**γ**-signalling in TRC and DC leading to NO production early after infection might not only control the expansion, but also the contraction phase of CD8+ T cells, such as by nitrosylating and thereby altering key proteins of the IL-2 or TCR signaling pathway [42], [45]. More experiments will be necessary to test this hypothesis and to understand the underlying mechanism.

Strikingly, iNOS induction and NO_2_^−^ production was highest when TRC, activated DC and IFN**γ** were present together suggesting only adjacent TRC become licensed to suppress T cell expansion with this being a highly controlled and transient process. The DC-derived signals leading to iNOS expression in TRC remain to be identified. Good candidates are IL-1α/β and TNFα which may act synergistically with IFN**γ**, similar to iNOS expression in MSC [36]. NO effects are known to be highly dose-dependent. Low NO doses can enhance T cell proliferation, as possibly suggested by the decreased T cell expansion in some of the BM chimera experiments (*Inos* ^−/−^ into wt). Higher NO doses are known to induce cell-cycle arrest and apoptosis of T cells [35], [46] indicating that the reduced expansion seen in presence of TRC may represent a combined effect of reduced proliferation and increased apoptosis of T cells. The dual role of NO may also explain the only two-fold higher expansion of T cells in the absence of iNOS. Given that NO is a highly reactive gas close proximity to TRC is likely to be critical in inhibiting T cell and DC activation. To further improve this process cytokine-stimulated TRC could actively recruit or retain primed T cells, as suggested for MSC [36]. Interestingly, TNFα-stimulated TRC were shown to express the IFN**γ**-inducible chemokine CXCL10 that attracts activated but not naïve T cells [47].

Both *in vitro* and *in vivo*, we found not only TRC but also DC to express iNOS and produce NO. It is possible that the iNOS^+^ DC subset identified *in vivo* represents the inflammatory TipDC (TNFα^+^iNOS^+^CCR2^+^) which are recruited into the splenic T zone early during infection with *Listeria monocytogenes* [48] or other pathogens [49] as they were also CD11c^int^ in our analysis (unpublished observation). Interestingly, increased T cell proliferation was also reported for *Ccr2* ^−/−^ mice lacking TipDC [48]. *In vitro* we found iNOS expression in BM-DC to be induced in a synergistic manner by IFN**γ** and TRC presence, similar to synergistic effects of IFN**γ** and BM-DC on iNOS expression by TRC. While the factors involved in the cellular crosstalk remain to be identified these findings point to the interdependence of these two cell types in inhibition of T cell expansion, an observation which needs further *in vivo* studies with cell-type specific *Inos* deletion. Based on our current results we propose the following working model (Fig. S7): Low levels of iNOS expression are induced in both immigrating DC and TRC when they meet in the T zone early during the immune response. This may increase the threshold required for T cell priming and thereby prevent the activation of low-affinity T cells that are often self-reactive. Once the first T cells are primed and produce IFN**γ** they may boost iNOS expression in both cell types in a transient fashion thereby inducing a negative feedback loop dampening T cell expansion by either slowing down their proliferation or diminishing their survival.

The immunosuppressive feature of mesenchymal cells is intriguing. It raises the question of why these cells have this function, especially when considering TRC localize to the T zone of SLO where inhibitory effects are less expected but well known from TipDC and regulatory T cells. We would like to propose two possible reasons: 1) Keeping the LN in a slightly suppressive state might be a mechanism to limit the activation of potentially auto-reactive cells while allowing strong immune responses to foreign antigens to occur. In line with this hypothesis we found *Cox2* transcripts to be highly expressed in naïve LN. Others showed that high iNOS and NO_2_^−^ levels are frequently associated with autoimmune disease and Th1-mediated inflammation [35], [46]. Consistent with a protective role of this pathway in certain autoimmune diseases, *Inos* ^−/−^ and *Ifng* ^−/−^ mice have a higher incidence, increased severity and less relapses of experimental autoimmune encephalitis that correlate with increased proliferation of auto-reactive T cells relative to WT mice [50], [51]. Similarly, in a mouse model for myasthenia gravis *Inos* ^−/−^ mice develop more self-reactive T and B cells, including epitope spreading [52]. 2) In contrast to recirculating hematopoietic cells fibroblasts resident in lymphoid and non-lymphoid organs have a function for the organ itself: ensure its integrity and functionality. During injury, such as infection or inflammation, fibroblasts perform tissue repair to reestablish homeostasis. LN swelling during immune response can be viewed as a threat to the organ as it is characterized by the dramatic increase in organ size due to the influx of many naïve lymphocytes as well as the rapid expansion of antigen-specific lymphocytes. The strong increase in lymphocyte numbers within the T zone may be harmful for the existing TRC network that functions as the structural platform for T cell priming and probably also T cell differentiation. Destruction of the TRC architecture, such as after LCMV infection, correlates well with immunodeficiency [6], [32], [53]. Limiting T cell expansion early in the immune response might be necessary to give TRC the time to adjust step-wise to the new space demands and to start proliferating thereby increasing the scaffold size accommodating T cells and DC. Such a process may ensure continuous functionality of the growing organ which is finally also to the benefit of efficient effector T cell generation.

## Materials and Methods

### Ethics Statement

This study was carried out in strict accordance with the Swiss act for animal welfare. All mouse experiments, including the protocols, were authorized by the Swiss Federal Veterinary Office (Bern, Switzerland) with permits issued to S.A.L. (number 1612.2), B.J.M. (number 2216) and D.Z. (number 2308). All efforts were made to minimize suffering.

### Mice and immunizations

Mice used were C57BL/6 (B6, CD45.2^+^); Janvier), B6 OT-I CD45.1^+^, *Ubiquitin-gfp* transgenic and *Inos^−/−^* (Jackson laboratories). For immunization mice were subcutaneously injected at six sites in the back to target six pLN. 25 µg NP-CGG (Biosearch Technology) was diluted in Montanide ISA 25 (25% in PBS; Seppic). Alternatively, 0.33×10^6^ pfu VSV-OVA [54] were injected after retro-orbitally grafting 0.1×10^6^ OT-I splenocytes. Bone marrow (BM)-chimeras: BM from donor-mice was obtained from femur and tibia by crushing bones with a mortar. 15×10^6^ BM cells were injected retro-orbitally into recipient mice irradiated twice with 450 rad in a 4 h interval. The following 4 weeks mice received the antibiotic ‘Baytril 10%’ (1/1000) in the drinking water. WT mice used were either CD45.2^+^ or Cd45.1^+^ while *Inos^−/−^* mice were CD45.1^+^. All mice were maintained in specific pathogen-free conditions.

### Flow cytometry

0.1–2×10^6^ cells were blocked (2% normal-mouse-serum (Sigma) or anti-CD16/32 antibody (2.4G2) and then stained with antibodies (see table S1) on ice. Dead cells were excluded using 7-AAD (7-Aminoactinomycin-D) or DAPI (4,6-diamidino-2-phenylindole), or Aqua (all from Invitrogen) in case of fixed cells. Intracellular staining: Cells were fixed with 4% PFA, pemeabilized using 0.1% saponin (Sigma) and incubated with anti-IFN**γ** (XMG1.2) (45 min) or anti-iNOS antibody (rabbit antibody; Millipore) followed by Alexa488-coupled donkey-anti-rabbit IgG (Molecular Probes) (30 min each). For IFN**γ** staining: cells were re-stimulated *in vitro* with 1 µM SIINFEKL peptide in the presence of Brefeldin-A (10 µg/ml, AppliChem) for 3–4 h at 37°C. Data were acquired on a FACSCanto or LSR II flow cytometer (both BectonDickinson) and were analyzed with FlowJo software (TreeStar).

### *Ex vivo* stromal cell isolation

LN from CO_2_-killed mice were dissected, their capsule opened with a 26-gauge needle and the organs digested for 30 min at 37°C in DMEM (Invitrogen) containing collagenase IV (3 mg/ml; Worthington), DNAse-I (40 mg/ml; Roche), 2.5% FCS and 1.2 mM CaCl_2_, 10 mM HEPES and 50 IU/ml Penicillin, 50 µg/ml streptomycin. Subsequently, EDTA was added (5 mM final) and remaining clumps dissolved by pipetting, passed through a 40-µm mesh and re-suspended in complete RPMI containing 10% FCS. For cell isolation from femoral muscle, kidney and heart, the respective organs were dissected, cut into small pieces and digested as described above for 1 h at 37°C. Cell isolation from ears: Ears were split with forceps and digested in 0.5% trypsin (Sigma) and 5 mM EDTA for 20 min at 37°C to separate dermal and epidermal sheets. These were cut into small pieces and digested for 2 h as described. After digestion stromal cells were enriched by panning using antibodies against CD45 (M1/9.4.3), CD31 (GC-51), CD11c (N418) and CD11b (M1/70). Cells were counted using trypan blue dye and an automated cell counter (Countess, Invitrogen).

### Cell lines and bone-marrow-derived dendritic cells (BM-DC)

Stromal cells were isolated as described above from various tissues of naïve B6 or *Ubiquitin-gfp* B6 mice. After overnight culture in complete RPMI non-adherent cells were washed away. Adherent cells were cultured until confluent, then split on new dishes. Cells were used between passage 5 and 25. Other B6-derived cell lines used: B16-F10 and MC-38 tumor lines (kindly provided by A.Donda, Lausanne) [55], spleen and lung fibroblasts (C.Buckley [26]), MEF (M.Heikenwalder, Munich) and MSC (P.Nelson, Munich [56]). B6 BM-DC were generated as previously described [57].

### T cell activation assay

Stromal cells: Per 24-well 1×10^4^ stromal cells from lines were seeded in complete RPMI and after overnight culture irradiated with 1000rad. Initially, experiments with non-irradiated or irradiated pLN2 were performed with no difference in the outcome (data not shown). *Ex vivo* stromal cells were isolated as described above and non-adherent cells washed away after overnight culture (density comparable with lines); they were non-proliferative and therefore not irradiated. BMDC: They were activated with 0.5 µg/ml LPS (Sigma) for 6 h at 37°C. 2 h after LPS addition 1 µM SIINFEKL peptide was added. 1×10^4^ BM-DC were added per 24-well. T cells: They were obtained ex vivo from spleen and pLN dissected from CO_2_-killed WT B6 and OT-I transgenic mice and suspended by meshing. Erythrocytes were removed using red blood cell lysis buffer (Tris-Ammonium chloride based). CD8 cells were enriched by panning using antibodies to B220 (RA3-6B2), CD4 (H129.19.6), CD11b (M1/70) and CD11c (N418). OT-I cells were labeled with 2 µM CFSE (Invitrogen). Only 50% of WT B6 T cells were CFSE-labeled to identify the peak of undivided cells, with the 50% unlabeled T cells showing the background fluorescence of T cells. Per 24-well 0.04×10^6^ CFSE^+^ OT-I cells together with 0.98×10^6^ unlabeled B6 WT cells and 0.98×10^6^ CFSE^+^ B6 WT cells were added. The assay was performed in complete RPMI enriched with 1×MEM (Invitrogen), 3 ng/ml murine IL-7 (Peprotech) and 10 U/ml human IL-2 (Merck Serono). After 2–4 days, cells were harvested, counted and analyzed by flow cytometry. Transwell assays used were 0.4 µm transwell chambers (HTS 24-well, Vitaris). Blocking experiments used 10 µM indomethacin (Sigma), 1 µM 1400W ( = dihydrochloride; Sigma), 10 µM 1-Methyl-L-tryptophan (1-MT; Sigma), 200 µM (S)-(2-Boronoethyl)-L-cysteine (BEC, Calbiochem), 10 µg/ml anti-PD-L1 (MIH5, eBioscience), 20 µg/ml anti-IL-10 (kind gift from F.Tacchini, Lausanne, Switzerland) or 30 µg/ml anti-TGFβ (clone 1D11.16.8; BioXCell). To better compare the decrease of T cell proliferation between experiments the attenuating effect by the stromal cells was defined as percent inhibition in divided OT-I T cell numbers relative to the ‘no stroma’ control: {1-(number of proliferated cells)_stroma_/(number of proliferated cells)_no stroma_}*100). The ‘no stroma’ control was considered as 0% inhibition; absence of proliferating OT-I cells was considered as 100% inhibition.

### T cell activation by anti-CD3 and anti-28 beads

2500 stromal cells were seeded per 96-well and after overnight culture irradiated with 1000 rad. 2.5×10^5^ T cells mixed with 1.25×10^5^ anti-CD3/28 beads (Dynabeads, Invitrogen) were added. After 2–3 days of co-culture cells were harvested by gentle pipetting. Live cells were counted and analyzed by flow cytometry.

### T cell activation by TRC-conditioned BM-DC

10'000 stromal cells were seeded per 24-well and after overnight culture 10'000 LPS-activated and SIINFEKL-pulsed BM-DC were added. After overnight culture BM-DC were separated by magnetic cell sorting (MACS): Cells were stained with biotinylated anti-gp38-antibody (clone 8.1.1) followed by streptavidin-coupled magnetic beads (Miltenyi). Cells were separated using MS-columns (Miltenyi) according to the manufactures instructions. Per 96-well 2500 TRC-conditioned-DC were co-cultured with 0.01×10^6^ CFSE labeled OT-I cells mixed with 0.48×10^6^ unspecific WT T cells for 3 days.

### T cell activation and cytokine stimulation of stromal cells in chamber slides

3000 stromal cells were seeded per well in 8-well chamber slides (Falcon) and cultured overnight. T cell activation: 0.25×10^6^ WT T cells and 0.125×10^6^ anti-CD3/28 beads were added or 5000 CFSE-labeled OT-I T cells mixed with 0.245×10^6^ WT T cells together with 5000 LPS-activated and SIINFEKL-pulsed BM-DC. Cytokine stimulation: IFN**γ** IL-1β, TNFα, LTα3 (all 10 ng/ml, from Peprotech), 500 U/ml IFNα (PBL, Interferon source) or the agonistic antibody against LTβR (4H8 WH2; provided by C.Ware) were added to stromal cells for 7 h or 24 h.

### In vitro cytotoxicity assay

Target cells (T): WT splenocytes were labeled with 0.16 µM or 0.5 µM eFluor670 (eBioscience). The eFluro670^high^ population was pulsed with 1 µM SIINFEKL for 1 h at 37°C. Per 96-well 5000 eFluor670^high^ and 5000 eFluor670^low^ cells were added. Effector cells (E) were harvested from the T cell activation assay on day 4 or from spleen or pLN from VSV-infected mice. Cells were mixed in different E/T ratios and after overnight culture analyzed by flow cytometry. Alternatively, they were isolated from homogenized spleen or pLN from VSV-infected mice. Effector cells were added to target cells in different ratios and incubated overnight. The ratio of target cells was analyzed by flow cytometry, as were the input numbers of OT-1 effector T cells allowing the calculation of the effective E/T ratio. Percentage specific lysis = {1−(specific survival)_sample_/(specific survival)_control_}*100 with specific survival = eFluor670^high^/eFluor^low^, (control: target cells only).

### Nitrite detection

NO_2_^−^ in cell culture supernatants was measured using the Griess assay. 0.1% N-1-naphtylethylenediamine dihydrochloride (Sigma) was mixed with p-aminobenzensulfonamide (Sigma) in 5%phosphoric acid; 100 µl of this mixture were incubated with 100 µl cell culture supernatant. After 10 min incubation at RT absorbance was measured at 550 nm and background absorbance at 690 nm was subtracted as well as the absorbance of complete RPMI. In each measurement a NO_2_^−^ -standard series (100-0.1 µM NaNO_2_; Sigma) was included.

### Immunofluorescence microscopy

Staining and microscopy of cryosections were performed as previously described [9]. Cells cultured in chamber slides: chambers were removed according to the manufactures description, staining and microscopy was performed as done for cryosections (for antibodies used see table S2). Images were acquired with a Zeiss Axioplan microscope at RT and treated with ImageJ (http://rsbweb.nih.gov/ij/) and Photoshop software (Adobe).

### Transcript analysis

RNA isolation reverse transcription and quantitative real-time PCR was performed as described [9]. For additional primers used see table S3. Efficiency-corrected expression of *Inos*, *Cox2*, *Ccl19* and *Ccl21* was normalized by dividing with the geometric mean of expression of two housekeeping genes.

### Statistical analysis

F-test was performed to determine usage of equal or unequal variance in the t-test, with *P*<0.05 considered as unequal variance. Statistical significance was determined with students t-test.

## Supporting Information

Figure S1 **Surface phenotype of TRC lines and ex vivo isolated TRC.** Flow cytometric analysis of the surface phenotype of TRC lines (**A**) or TRC isolated *ex vivo* from pLN (**B**) used in the T cell activation assay shown in Figure 1. (**A**) The first row shows dot plots of CD31 versus CD45 expression and the percentage of CD45^−^ CD31^−^ cells. (± standard deviation). Following rows show histograms with the indicated surface markers on cells pregated on CD45^−^ CD31^−^ cells. pLN2 and pLN1 are two different TRC lines derived from pLN-pools derived from distinct mice. pLN2 cells also express CD54 (ICAM-1), CD140b (PDGF-Rβ) and CD105 (Endoglin) (not shown). (**B**) CD45^−^ CD31^−^ cells were analyzed in dot plots for CD31 versus gp38 expression (first row). The following rows show the indicted surface markers on CD45^−^ CD31^−^ CD35^−^ gp38^+^ cells (lower quadrant on the right in the gp38 versus CD31 dot plot; displayed as black line). Grey shadowed curves show the ‘no primary antibody’ control. (**A,B**) representative for ≥3 independent experiments.
(TIF)

Figure S2 **Surface phenotype of non-lymphoid cell lines and** ***ex vivo*** **isolated stromal cells.** Flow cytometric analysis of the surface phenotype of (**A**) non-lymphoid cell lines or (**B**) *ex vivo* stromal cells isolated from dermis, epidermis and kidney, and used in the T cell activation assay shown in Figure 3. (**A**) The first row shows dot plots of CD31 versus CD45 expression on the cell lines, the following rows show histograms with the indicted surface markers on CD45^−^ CD31^−^ cells. (**B**) CD45^−^ CD35^−^ cells were analyzed in dot plots for CD31 versus gp38 expression (first row). The following rows show histograms with the indicated surface markers on CD45^−^ CD35^−^ CD31^−^ gp38^+^ cells as black line, and gp38^−^ CD31^−^ cells as dotted line (includes epithelial cells. Grey shadowed curves show the ‘no primary antibody‘ control. (**A,B**) representative for 2–3 independent experiments.
(TIF)

Figure S3 **Suppression of T cell proliferation by TRC is not mediated by IDO, PD-L1, TGFβ, IL-10 or arginase-1.** Flow cytometric analysis of the T cell activation assay (as described in Fig. 1) in the presence of pharmacological inhibitors of IDO (10 µM 1-MT), arginase-1 (200 µM BEC) or blocking antibodies against PD-L1 (10 µg/ml), TGFβ (30 µg/ml) or IL-10 (20 µg/ml). One out of several tested inhibitor concentrations is shown along with the percentage of inhibition (as in Fig. 1). The dotted line shows the average inhibition by pLN2 TRC without inhibitor/blocking antibodies. n≥3, representative for 2–3 experiments.
(TIF)

Figure S4 **IFNγ induces iNOS expression and NO production in ex vivo TRC, while stimulation of pLN2 with different cytokines does not induce** ***Cox2*** **,** ***Ccl21*** **or** ***Ccl19*** **transcription.** (**A**) Immunohistological analysis of iNOS protein expression in *ex vivo* TRC. TRC-enriched cells from pLN of WT mice were cultured for 2 days either alone (top row), in the presence of WT T cells without (second row) or with anti-CD3/28 beds (third row) or in presence of 10 ng/ml recombinant IFN**γ** (last two rows). For the first four rows the first column shows iNOS expression (red) and DAPI+ nuclei (blue), while for the last row, the first column show the ‘no primary antibody control’ (red), the second column shows gp38^+^ CD31^−^ TRC and gp38^+^ CD31^+^ lymphatic cells. Arrows show examples of gp38^+^ CD31^−^ TRC expressing iNOS. Scale bar: 50 µm. (**B**) Analysis of NO_2_− concentration in the supernatants from the co-cultures shown in (**A**). (**C**) Quantitative RT-PCR analysis of *Cox2*, *CCl21* and *CCl19* mRNA levels in pLN2 stimulated with various cytokines or agonistic antibody to LTβR (αLTβR) for 7 h or 24 h. The relative expression levels are shown. Different symbols indicate different experiments. (**A–C**) n = 3–4, (**A–C**) respresentative for 1–2 experiments. * p<0.05,**p<0.0,***p<0.001, p values are relative to unstimulated pLN2.
(TIF)

Figure S5 ***Inos*** **expression and CD8+ T cell effector functions in VSV-OVA infected WT or** ***Inos*** **^−/−^ mice.** WT or *Inos* ^−/−^ mice were retro-orbitally grafted with 100'000 splenocytes from OT-I transgenic mice one day prior to subcutaneous infection with VSV-OVA. (**A**) Regional pLN from infected mice were harvested 1, 2 or 4 days after infection and analyzed for *Inos* mRNA by by quantitative real time-PCR on crude fractions of LN. Normalized *Inos* mRNA levels are shown. (**B, C**) Draining pLN and spleen were harvested on day 4 and 6 after infection, homogenized and analyzed for intracellular IFN**γ** protein expression in OT-I T cells using flow cytometry (**B**) or for killing activity (**C**). (**B**) Histograms show the percentage of OT-I T cells (± standard deviation) expressing IFN**γ**. Bar graphs show the median fluorescence intensity of the IFN**γ** staining within OT-I T cells. (**C**) Cells from day 6 were used in an *in vitro* killing assay. The specific lysis of target cells is shown for the respective effector to target ratios. (**A–C**) n = 3 (**A,B**) representative for 2 experiments, (**C**) 1 experiment (similiar data were obtained for day 8 LN and spleen, not shown).
(TIF)

Figure S6 **Bone marrow chimeras lacking** ***Inos*** **in the non-hematopoietic system show a trapping defect in immunized lymph nodes.** To assess the relative contribution of hematopoietic versus non-hematopoietic cells as iNOS source, BM chimeras were generated having Inos-deficiency in either the hematopoietic system (*Inos* ^−/−^ BM into WT hosts) or non-hematopoietic system (WT in *Inos* ^−/−^ and were compared with control chimeras (WT into WT). 2 months after reconstitution, BM-chimeras were infected with VSV-OVA (as described in Figure 7) and 4 days after infection pLN were collected, single cell suspensions counted and stained before analysis using flow cytometry. As comparison non-immunized BM-chimeras are shown. *Inos* ^−/−^ into *Inos* ^−/−^ chimeras have not been made. Surprisingly, none of the chimeras showed an increased OT-I expansion. Rather, *Inos* -deficiency in either the hematopoietic or the non-hematopoietic system led to a strong decreased OT-I expansion. Surprisingly, lymphocyte trapping did not occur in the case of *Inos* -deficiency in the non-hematopoietic compartment, in contrast to the other two groups and the non-chimeric *Inos* ^−/−^ mice (Figure 7). Therefore, the expansion of OT-I T cells cannot be interpreted for that group of mice. The lack of OT-I expansion in the group of mice having *Inos* deleted in the hematopoietic system indicates also a positive role of *Inos* in T cell proliferation, presumably in a low but not high concentration, as previously suggested [46]. In all BM chimeras the chimerism was >85% as assessed by measuring ratio's of CD45.2 (WT) versus CD45.1 (*Inos* ^−/−^ or WT) expression on total LN cells using flow cytometry. 2–4 mice were in each group.
(TIF)

Figure S7 **Model showing how inflammatory cytokines may induce iNOS expression in TRC and create a negative feedback loop limiting antigen-specific T cell expansion.** During the early phase of immune response antigen-specific CD8+ T cells interact with antigen-bearing DC within the context of the TRC network within the T zone of the draining LN. Upon prolonged cognate interaction T cells start to produce IFN**γ** and possibly other cytokines that induce strong but transient iNOS expression in neighboring TRC as well as DC. The local production of NO creates a negative feedback loop in which NO and possibly other inhibitory factors limit the expansion of neighboring antigen-specific T cells. This effect is due in part to direct inhibition of T cell proliferation or survival, in part to a decrease in the stimulatory capacity of DC. NO is known to lead to nitrosylation of cysteine- and tyrosine-containing proteins thereby altering their function, including in T cells where reduced T cell proliferation was reported in presence of NO [35]. High NO concentrations may also reduce T cell survival. An alternative model is that *in vivo* innate immune cells, such as NK cells, become activated early during the response and release IFN**γ** that induces iNOS in TRC and DC. Together, these processes may prevent overshooting antigen-specific T cell expansion while affecting much less T cell differentiation. This selective negative regulation of T cell numbers by TRC and DC may allow gradual organ and stromal cell growth thereby achieving a compromise between preservation of functional organ structure and fast effector T cell differentiation. It is reminiscent of the role of TRC in controlling naïve T cell numbers [9], [13]. The early and transient induction of iNOS may have also an impact on later aspects of the immune response, such as the contraction phase and memory T cell generation, as they are thought to be controlled by the conditions encountered during the T cell priming phase [1], [44].
(TIF)

Table S1 **Staining reagents used for flow cytometry.**
(DOCX)

Table S2 **Staining reagents used for immunofluorescence microscopy.**
(DOCX)

Table S3 **Primer sequences for quantitative PCR analysis.**
(DOCX)
