# Supplementary figures and images for: Fibroblastic Reticular Cells From Lymph Nodes Attenuate T Cell Expansion by Producing Nitric Oxide
Source: PLoS One. 2011 Nov 14;6(11):e27618. doi: 10.1371/journal.pone.0027618 (PMC3215737; doi:10.1371/journal.pone.0027618)

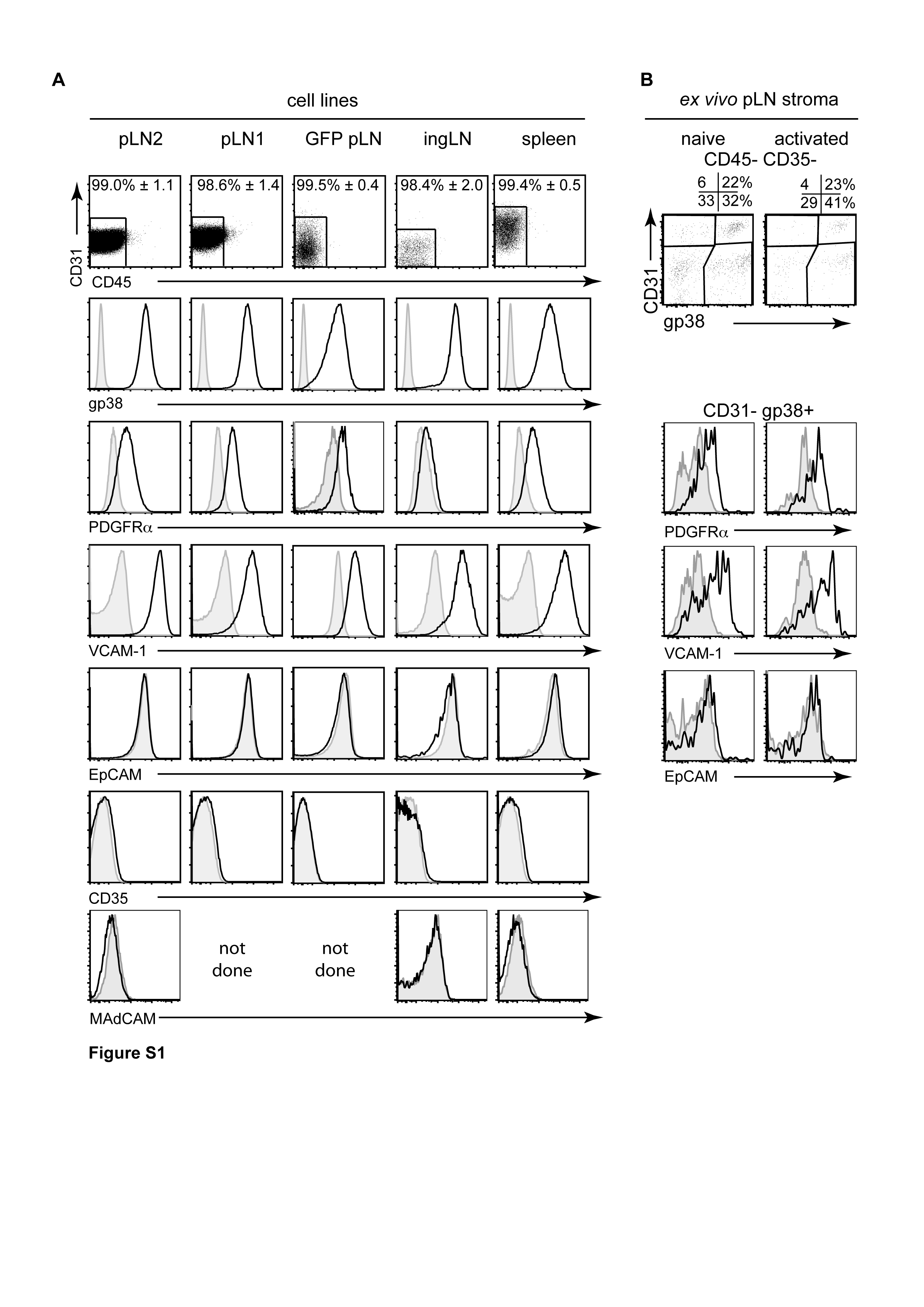

Supplement: Figure S1 — Surface phenotype of TRC lines and ex vivo isolated TRC. Flow cytometric analysis of the surface phenotype of TRC lines (A) or TRC isolated ex vivo from pLN (B) used in the T cell activation assay shown in Figure 1. (A) The first row shows dot plots of CD31 versus CD45 expression and the percentage of CD45− CD31− cells. (± standard deviation). Following rows show histograms with the indicated surface markers on cells pregated on CD45− CD31− cells. pLN2 and pLN1 are two different TRC lines derived from pLN-pools derived from distinct mice. pLN2 cells also express CD54 (ICAM-1), CD140b (PDGF-Rβ) and CD105 (Endoglin) (not shown). (B) CD45− CD31− cells were analyzed in dot plots for CD31 versus gp38 expression (first row). The following rows show the indicted surface markers on CD45− CD31− CD35− gp38+ cells (lower quadrant on the right in the gp38 versus CD31 dot plot; displayed as black line). Grey shadowed curves show the ‘no primary antibody’ control. (A,B) representative for ≥3 independent experiments. (TIF) [file pone.0027618.s001.tif]

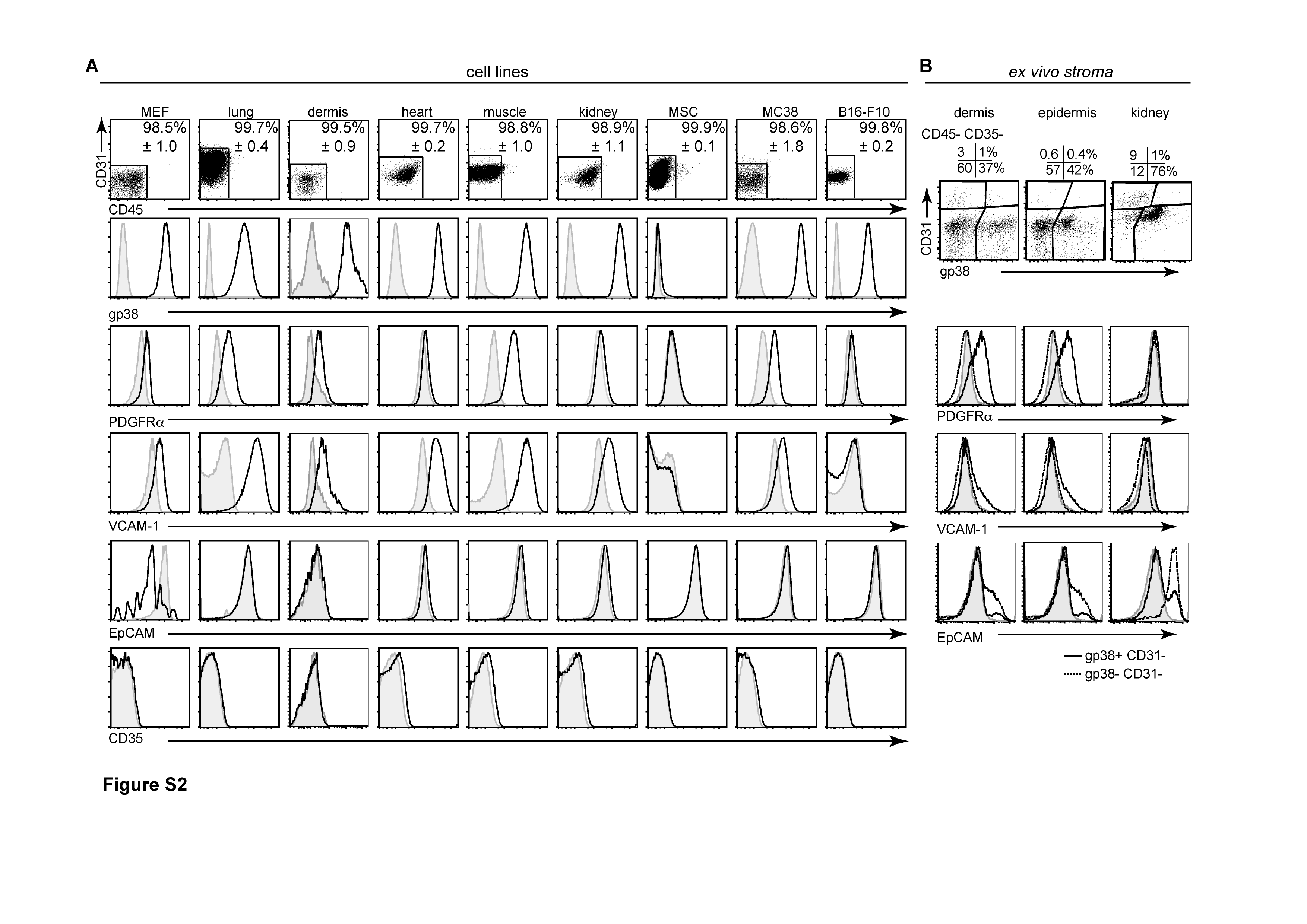

Supplement: Figure S2 — Surface phenotype of non-lymphoid cell lines and ex vivo isolated stromal cells. Flow cytometric analysis of the surface phenotype of (A) non-lymphoid cell lines or (B) ex vivo stromal cells isolated from dermis, epidermis and kidney, and used in the T cell activation assay shown in Figure 3. (A) The first row shows dot plots of CD31 versus CD45 expression on the cell lines, the following rows show histograms with the indicted surface markers on CD45− CD31− cells. (B) CD45− CD35− cells were analyzed in dot plots for CD31 versus gp38 expression (first row). The following rows show histograms with the indicated surface markers on CD45− CD35− CD31− gp38+ cells as black line, and gp38− CD31− cells as dotted line (includes epithelial cells. Grey shadowed curves show the ‘no primary antibody‘ control. (A,B) representative for 2–3 independent experiments. (TIF) [file pone.0027618.s002.tif]

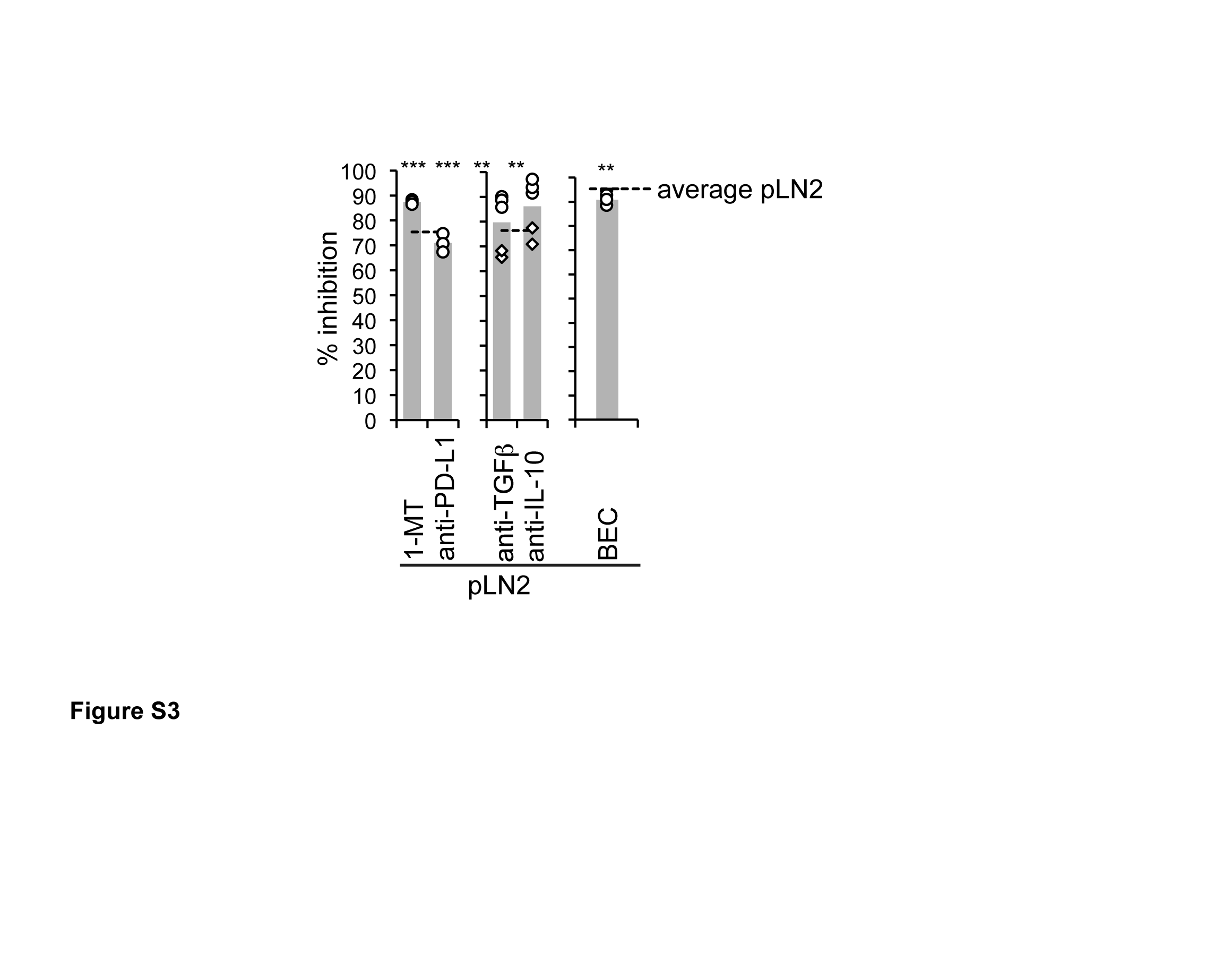

Supplement: Figure S3 — Suppression of T cell proliferation by TRC is not mediated by IDO, PD-L1, TGFβ, IL-10 or arginase-1. Flow cytometric analysis of the T cell activation assay (as described in Fig. 1) in the presence of pharmacological inhibitors of IDO (10 µM 1-MT), arginase-1 (200 µM BEC) or blocking antibodies against PD-L1 (10 µg/ml), TGFβ (30 µg/ml) or IL-10 (20 µg/ml). One out of several tested inhibitor concentrations is shown along with the percentage of inhibition (as in Fig. 1). The dotted line shows the average inhibition by pLN2 TRC without inhibitor/blocking antibodies. n≥3, representative for 2–3 experiments. (TIF) [file pone.0027618.s003.tif]

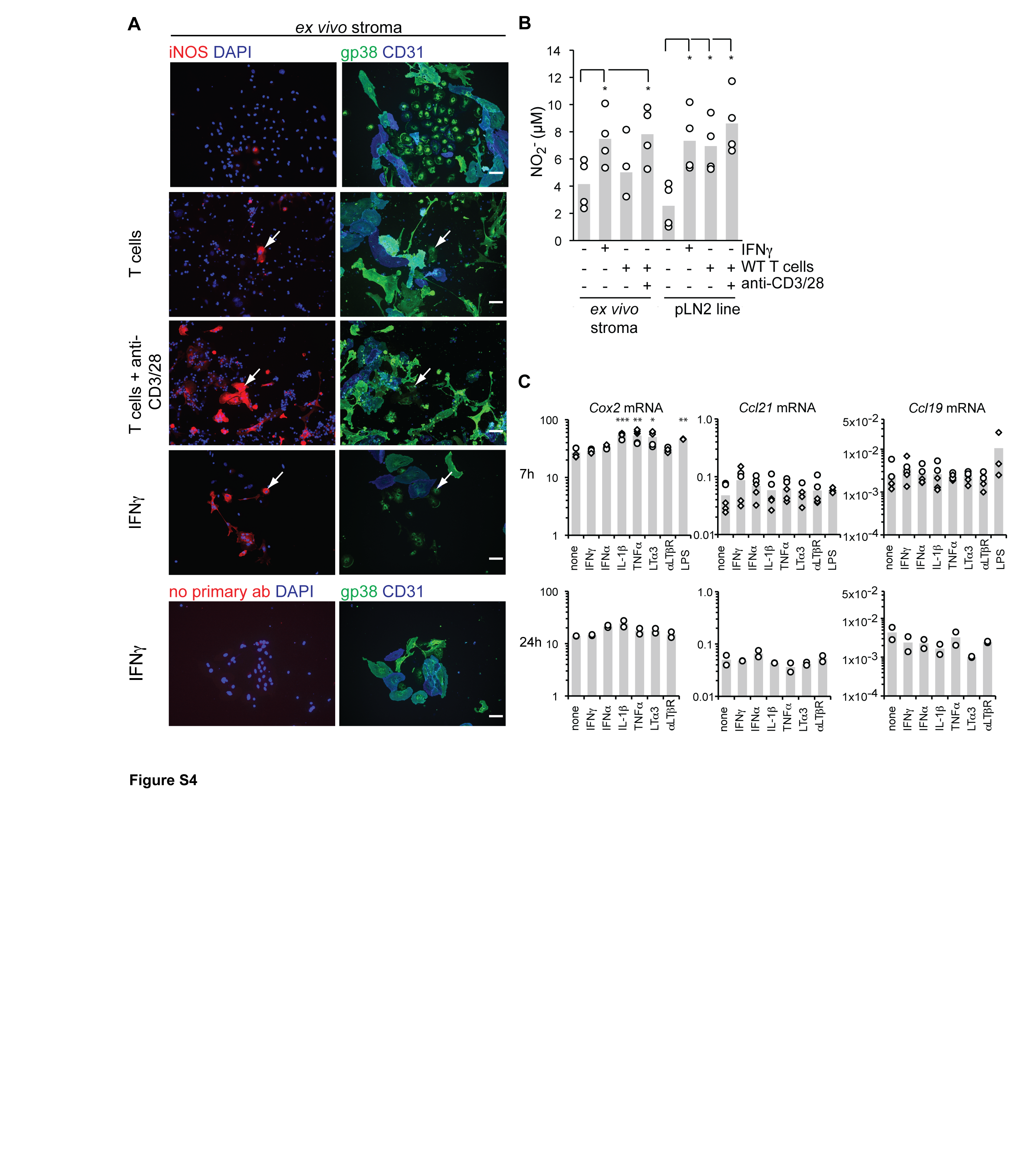

Supplement: Figure S4 — IFNγ induces iNOS expression and NO production in ex vivo TRC, while stimulation of pLN2 with different cytokines does not induce Cox2 , Ccl21 or Ccl19 transcription. (A) Immunohistological analysis of iNOS protein expression in ex vivo TRC. TRC-enriched cells from pLN of WT mice were cultured for 2 days either alone (top row), in the presence of WT T cells without (second row) or with anti-CD3/28 beds (third row) or in presence of 10 ng/ml recombinant IFNγ (last two rows). For the first four rows the first column shows iNOS expression (red) and DAPI+ nuclei (blue), while for the last row, the first column show the ‘no primary antibody control’ (red), the second column shows gp38+ CD31− TRC and gp38+ CD31+ lymphatic cells. Arrows show examples of gp38+ CD31− TRC expressing iNOS. Scale bar: 50 µm. (B) Analysis of NO2− concentration in the supernatants from the co-cultures shown in (A). (C) Quantitative RT-PCR analysis of Cox2, CCl21 and CCl19 mRNA levels in pLN2 stimulated with various cytokines or agonistic antibody to LTβR (αLTβR) for 7 h or 24 h. The relative expression levels are shown. Different symbols indicate different experiments. (A–C) n = 3–4, (A–C) respresentative for 1–2 experiments. * p<0.05,**p<0.0,***p<0.001, p values are relative to unstimulated pLN2. (TIF) [file pone.0027618.s004.tif]

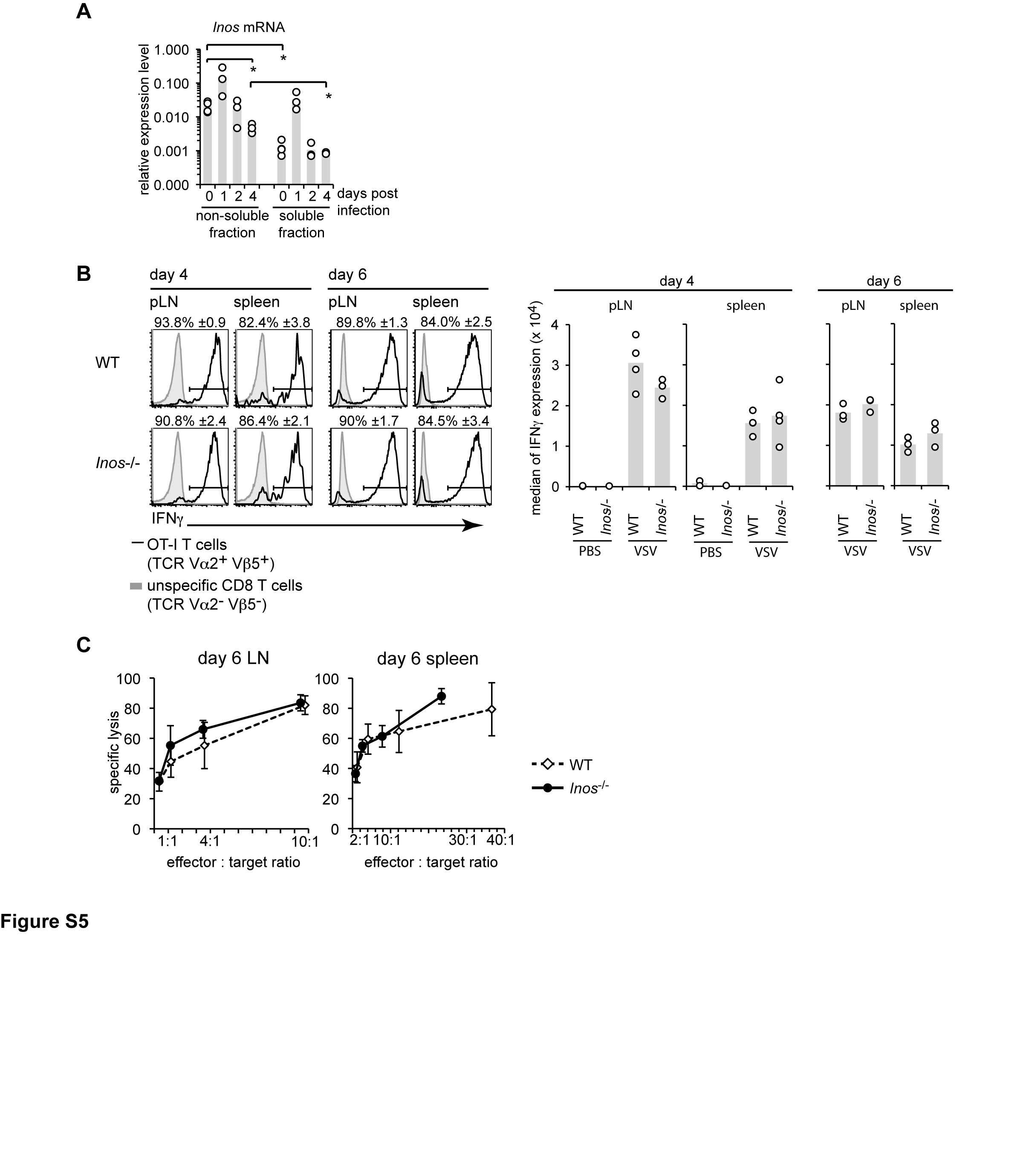

Supplement: Figure S5 — Inos expression and CD8+ T cell effector functions in VSV-OVA infected WT or Inos −/− mice. WT or Inos −/− mice were retro-orbitally grafted with 100'000 splenocytes from OT-I transgenic mice one day prior to subcutaneous infection with VSV-OVA. (A) Regional pLN from infected mice were harvested 1, 2 or 4 days after infection and analyzed for Inos mRNA by by quantitative real time-PCR on crude fractions of LN. Normalized Inos mRNA levels are shown. (B, C) Draining pLN and spleen were harvested on day 4 and 6 after infection, homogenized and analyzed for intracellular IFNγ protein expression in OT-I T cells using flow cytometry (B) or for killing activity (C). (B) Histograms show the percentage of OT-I T cells (± standard deviation) expressing IFNγ. Bar graphs show the median fluorescence intensity of the IFNγ staining within OT-I T cells. (C) Cells from day 6 were used in an in vitro killing assay. The specific lysis of target cells is shown for the respective effector to target ratios. (A–C) n = 3 (A,B) representative for 2 experiments, (C) 1 experiment (similiar data were obtained for day 8 LN and spleen, not shown). (TIF) [file pone.0027618.s005.tif]

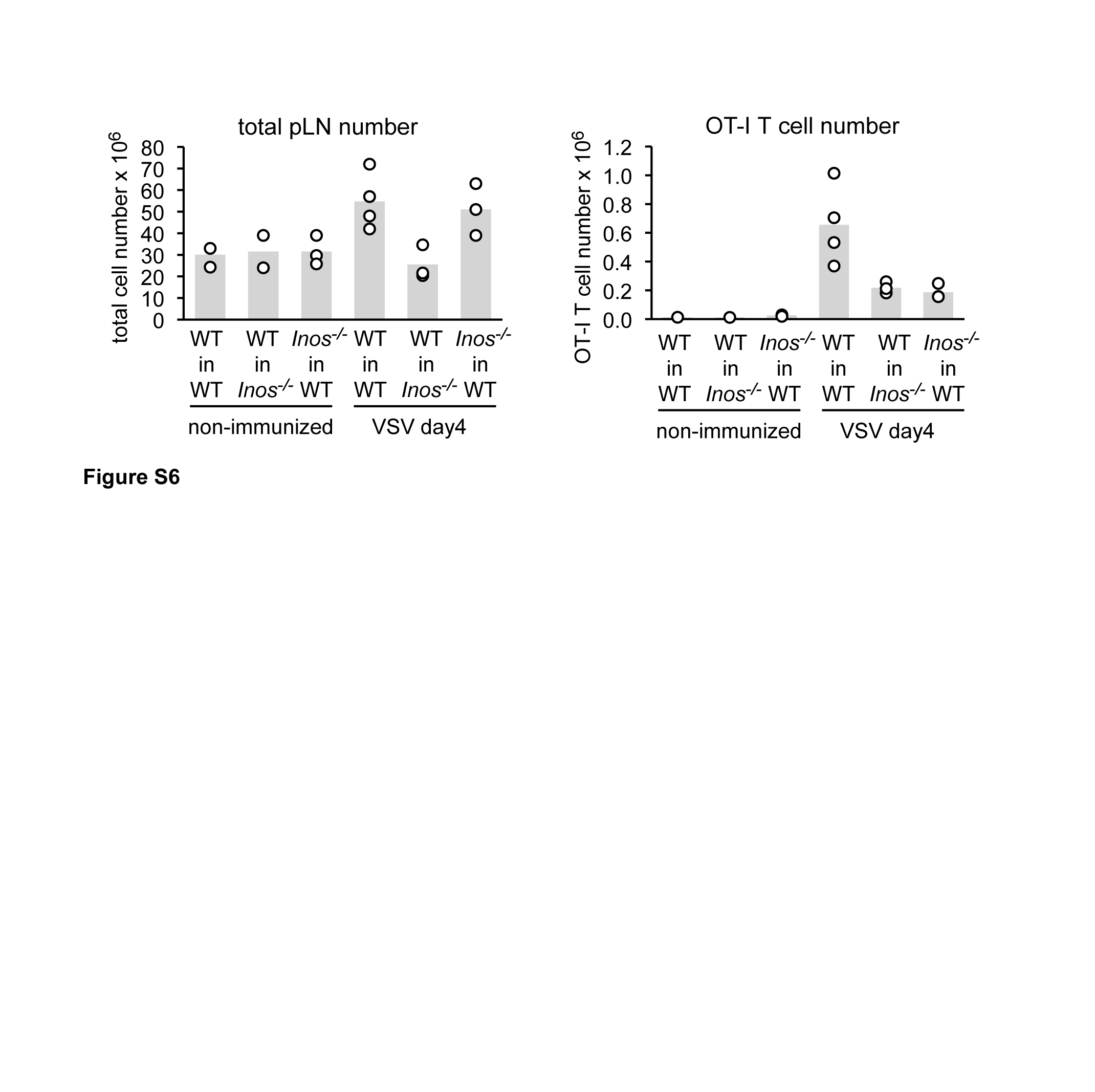

Supplement: Figure S6 — Bone marrow chimeras lacking Inos in the non-hematopoietic system show a trapping defect in immunized lymph nodes. To assess the relative contribution of hematopoietic versus non-hematopoietic cells as iNOS source, BM chimeras were generated having Inos-deficiency in either the hematopoietic system (Inos −/− BM into WT hosts) or non-hematopoietic system (WT in Inos −/− and were compared with control chimeras (WT into WT). 2 months after reconstitution, BM-chimeras were infected with VSV-OVA (as described in Figure 7) and 4 days after infection pLN were collected, single cell suspensions counted and stained before analysis using flow cytometry. As comparison non-immunized BM-chimeras are shown. Inos −/− into Inos −/− chimeras have not been made. Surprisingly, none of the chimeras showed an increased OT-I expansion. Rather, Inos -deficiency in either the hematopoietic or the non-hematopoietic system led to a strong decreased OT-I expansion. Surprisingly, lymphocyte trapping did not occur in the case of Inos -deficiency in the non-hematopoietic compartment, in contrast to the other two groups and the non-chimeric Inos −/− mice (Figure 7). Therefore, the expansion of OT-I T cells cannot be interpreted for that group of mice. The lack of OT-I expansion in the group of mice having Inos deleted in the hematopoietic system indicates also a positive role of Inos in T cell proliferation, presumably in a low but not high concentration, as previously suggested [46]. In all BM chimeras the chimerism was >85% as assessed by measuring ratio's of CD45.2 (WT) versus CD45.1 (Inos −/− or WT) expression on total LN cells using flow cytometry. 2–4 mice were in each group. (TIF) [file pone.0027618.s006.tif]

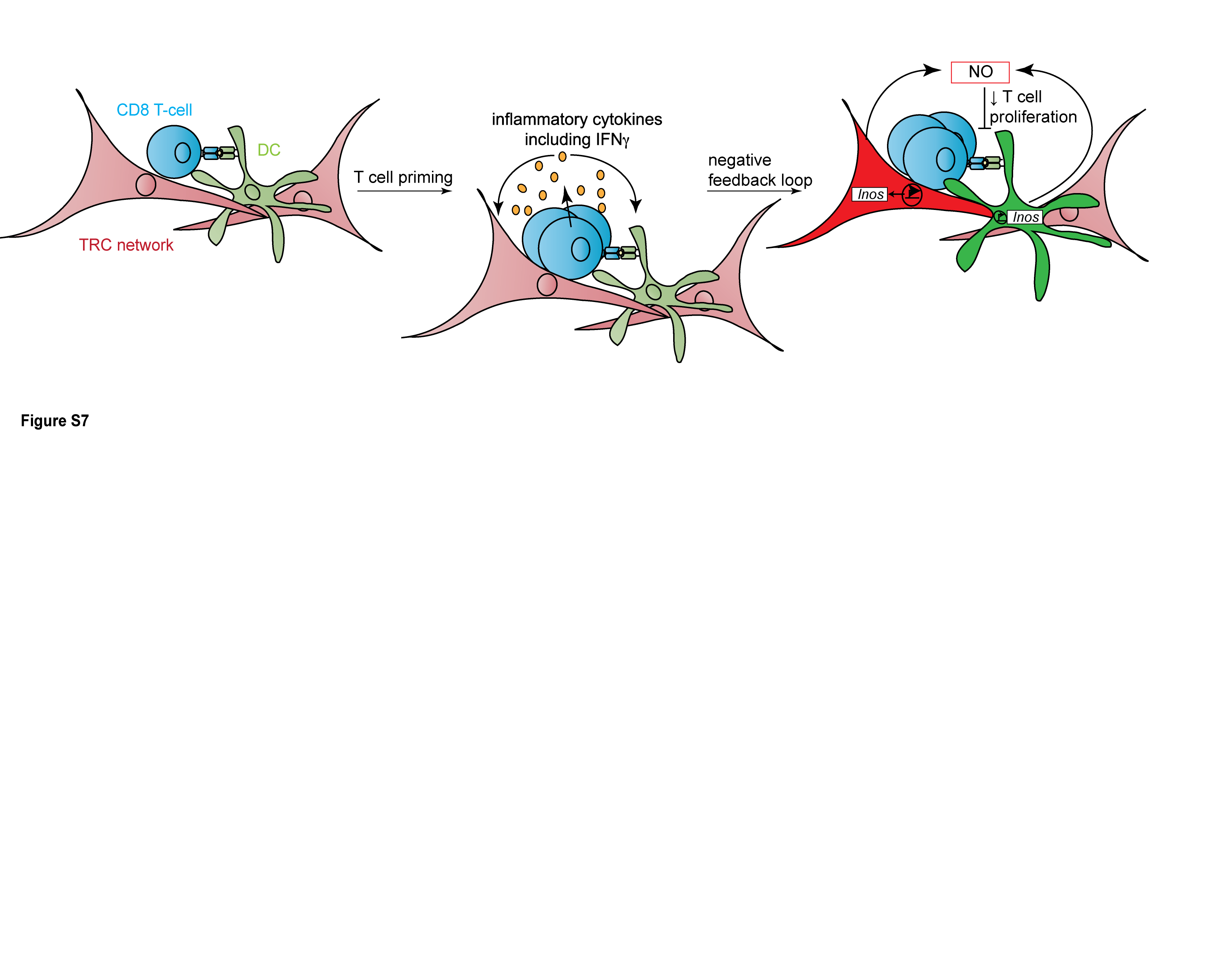

Supplement: Figure S7 — Model showing how inflammatory cytokines may induce iNOS expression in TRC and create a negative feedback loop limiting antigen-specific T cell expansion. During the early phase of immune response antigen-specific CD8+ T cells interact with antigen-bearing DC within the context of the TRC network within the T zone of the draining LN. Upon prolonged cognate interaction T cells start to produce IFNγ and possibly other cytokines that induce strong but transient iNOS expression in neighboring TRC as well as DC. The local production of NO creates a negative feedback loop in which NO and possibly other inhibitory factors limit the expansion of neighboring antigen-specific T cells. This effect is due in part to direct inhibition of T cell proliferation or survival, in part to a decrease in the stimulatory capacity of DC. NO is known to lead to nitrosylation of cysteine- and tyrosine-containing proteins thereby altering their function, including in T cells where reduced T cell proliferation was reported in presence of NO [35]. High NO concentrations may also reduce T cell survival. An alternative model is that in vivo innate immune cells, such as NK cells, become activated early during the response and release IFNγ that induces iNOS in TRC and DC. Together, these processes may prevent overshooting antigen-specific T cell expansion while affecting much less T cell differentiation. This selective negative regulation of T cell numbers by TRC and DC may allow gradual organ and stromal cell growth thereby achieving a compromise between preservation of functional organ structure and fast effector T cell differentiation. It is reminiscent of the role of TRC in controlling naïve T cell numbers [9], [13]. The early and transient induction of iNOS may have also an impact on later aspects of the immune response, such as the contraction phase and memory T cell generation, as they are thought to be controlled by the conditions encountered during the T cell priming phase [1], [44]. (TIF) [file pone.0027618.s007.tif]
