# Supplementary material for: Fibroblastic Reticular Cells From Lymph Nodes Attenuate T Cell Expansion by Producing Nitric Oxide
Source: PLoS One. 2011 Nov 14;6(11):e27618. doi: 10.1371/journal.pone.0027618 (PMC3215737; doi:10.1371/journal.pone.0027618)
Supplement: Table S1 — Staining reagents used for flow cytometry. (DOCX) [file pone.0027618.s008.docx]

| **Primary reagents** | | | | |
| --- | --- | --- | --- | --- |
| Target | species | Clone or designation | conjugate | Supplier |
| CD105 (Endoglin) | Rat | MJ7/18 | FITC | Hybridoma |
| CD106 (VCAM-1) | Rat | 429 | eFluor450 or biotin | eBioscience |
| CD11b | Rat | M1/70 | Alexa700 | eBioscience |
| CD11c | Rat | N418 | PE-Cy5.5 | eBioscience |
| CD140a (PDGFRα) | Rat | APA5 | FITC | Hybridoma |
| CD140b (PDGFRβ) | Rat | APB5 | FITC | Hybridoma |
| CD19 | Rat | ID3 | FITC | Hybridoma |
| CD25 | Rat | PC61 | Biotin | eBioscience |
| CD31 (PECAM1) | Rat | 390 | PE or PerCP-eFluor710 | BioLegend/eBioscience |
| CD326 (EpCAM) | Rat | G8.8 | Biotin | BioLegend |
| CD35/21 | Rat | 7E9 | PE-Cy7 | BioLegend |
| CD4 | Rat | RM-4-6 | PE-Cy5.5 | eBioscience |
| CD40 | Rat | FGK-45 | FITC | Hybridoma |
| CD44 | Rat | IM7 | Biotin | eBioscience |
| CD45 | Rat | 30-F11 | PE-Cy7 | eBioscience |
| CD45.1 | Mouse | A20.1 | Alexa647 | hybridoma |
| CD45R (B220) | Rat | RA3-6B2 | PE-Texas Red | BD Pharmingen |
| CD54 (ICAM-1) | Rat | YN1/1.7.4 | FITC or biotin | Hybridoma |
| CD62L (L-selectin) | Rat | MEL-14 | Alexa700 | eBioscience |
| CD80 (B7-1) | Armenian Hamster | 16-10A1 | Biotin | BioLegend |
| CD86 (B7-2) | Rat | GL-1 | PE | eBioscience |
| CD8α | Rat | 53-6.7 | PE-Cy7 | BioLegend |
| IFNγ | Rat | XMG1.2 | PE | eBioscience |
| iNOS | Rabbit | Polyclonal, cat nbr. 06-573 | Purified | Millipore |
| gp38 (podoplanin) | Syrian hamster | 8.1.1 | Alexa647 | Hybridoma |
| LYVE-1 (used as isotype control for iNOS) | Rabbit | Polyclonal | Purified | RELIATech |
| MAdCAM-1 | Rat | Meca-89 | FITC | Hybridoma |
| MHCI-SIINFEKL | Mouse | eBio25-D1.16 | PE | eBioscience |
| MHCII I-A/I-E | Rat | M5/114.15.2 | Alexa647 | Hybridoma |
| TCR Vα2 | Rat | B20.1 | FITC | Hybridoma |
| TCR Vβ5 | Mouse | MR9-4 | PE | BD Pharmingen |
| **Secondary reagents** | | | | |
| Streptavidin |  |  | PE | eBioscience |
| Streptavidin |  |  | APC-eFluor780 | eBioscience |
| Rabbit IgG | donkey |  | Alexa488 | Molecular Probes |

**Table S1: Staining reagents used for flow cytometry**
