# Supplementary material for: Fibroblastic Reticular Cells From Lymph Nodes Attenuate T Cell Expansion by Producing Nitric Oxide
Source: PLoS One. 2011 Nov 14;6(11):e27618. doi: 10.1371/journal.pone.0027618 (PMC3215737; doi:10.1371/journal.pone.0027618)
Supplement: Table S2 — Staining reagents used for immunofluorescence microscopy. (DOCX) [file pone.0027618.s009.docx]

**Table S2: Staining reagents used for immunofluorescence microscopy.**

| **Primary reagents** | | | | |
| --- | --- | --- | --- | --- |
| Target | species | Clone or designation | conjugate | Supplier |
| iNOS | Rabbit | Polyclonal, catalogue number 06-573 | purified | Millipore |
| gp38 (podoplanin) | Syrian hamster | 8.1.1 | purified | Hybridoma |
| CD31 | Rat | GC-51 | purified | Hybridoma |
| Secondary reagents | | | | |
| Rat IgG | Donkey |  | APC | Jackson Immunoresearch |
| Syrian Hamster IgG | Goat |  | biotin | Jackson Immunoresearch |
| Rabbit IgG | Donkey |  | Cy3 | Jackson Immunoresearch |
| Streptavidin |  |  | Alexa488 | Molecular Probes |
