# Supplementary material for: Fibroblastic Reticular Cells From Lymph Nodes Attenuate T Cell Expansion by Producing Nitric Oxide
Source: PLoS One. 2011 Nov 14;6(11):e27618. doi: 10.1371/journal.pone.0027618 (PMC3215737; doi:10.1371/journal.pone.0027618)
Supplement: Table S3 — Primer sequences for quantitative PCR analysis. (DOCX) [file pone.0027618.s010.docx]

**Table S3: Primer sequences for quantitative PCR analysis.**

| **Gene** | **Forward** | **reverse** |
| --- | --- | --- |
| *Inos* | gttctcagcccaacaatacaaga | gtggacgggtcgatgtcac |
| *Cox2* | tggtgcctggtctgatgatg | gtggtaaccgctcaggtgttg |
